# Empirical assessment of the assumptions of ComBat with diffusion tensor imaging

**DOI:** 10.1117/1.JMI.11.2.024011

**Published:** 2024-04-17

**Authors:** Michael E. Kim, Chenyu Gao, Leon Y. Cai, Qi Yang, Nancy R. Newlin, Karthik Ramadass, Angela Jefferson, Derek Archer, Niranjana Shashikumar, Kimberly R. Pechman, Katherine A. Gifford, Timothy J. Hohman, Lori L. Beason-Held, Susan M. Resnick, Stefan Winzeck, Kurt G. Schilling, Panpan Zhang, Daniel Moyer, Bennett A. Landman

**Affiliations:** a Vanderbilt University, Department of Computer Science, Nashville, Tennessee, United States; b Vanderbilt University, Department of Electrical Engineering, Nashville, Tennessee, United States; c Vanderbilt University, Department of Biomedical Engineering, Nashville, Tennessee, United States; d Vanderbilt University, Medical Scientist Training Program, Nashville, Tennessee, United States; e Vanderbilt University Medical Center, Vanderbilt Memory and Alzheimer’s Center, Nashville, Tennessee, United States; f Vanderbilt University Medical Center, Department of Medicine, Nashville, Tennessee, United States; g Vanderbilt University Medical Center, Department of Neurology, Nashville, Tennessee, United States; h Vanderbilt University Medical Center, Vanderbilt Genetics Institute, Nashville, Tennessee, United States; i National Institutes of Health, National Institute on Aging, Laboratory of Behavioral Neuroscience, Baltimore, Maryland, United States; j Imperial College London, Department of Computing, BioMedIA Group, London, United Kingdom; k Vanderbilt University Medical Center, Department of Radiology, Nashville, Tennessee, United States; l Vanderbilt University Medical Center, Department of Biostatistics, Nashville, Tennessee, United States; m Vanderbilt University Institute of Imaging Science, Nashville, Tennessee, United States

**Keywords:** diffusion tensor imaging, magnetic resonance imaging, ComBat, harmonization, bootstrap

## Abstract

**Purpose:**

Diffusion tensor imaging (DTI) is a magnetic resonance imaging technique that provides unique information about white matter microstructure in the brain but is susceptible to confounding effects introduced by scanner or acquisition differences. ComBat is a leading approach for addressing these site biases. However, despite its frequent use for harmonization, ComBat’s robustness toward site dissimilarities and overall cohort size have not yet been evaluated in terms of DTI.

**Approach:**

As a baseline, we match N=358 participants from two sites to create a “silver standard” that simulates a cohort for multi-site harmonization. Across sites, we harmonize mean fractional anisotropy and mean diffusivity, calculated using participant DTI data, for the regions of interest defined by the JHU EVE-Type III atlas. We bootstrap 10 iterations at 19 levels of total sample size, 10 levels of sample size imbalance between sites, and 6 levels of mean age difference between sites to quantify (i) $\beta_{\mathrm{AGE}}$, the linear regression coefficient of the relationship between FA and age; (ii) γ^sf*, the ComBat-estimated site-shift; and (iii) δ^sf*, the ComBat-estimated site-scaling. We characterize the reliability of ComBat by evaluating the root mean squared error in these three metrics and examine if there is a correlation between the reliability of ComBat and a violation of assumptions.

**Results:**

ComBat remains well behaved for $\beta_{\mathrm{AGE}}$ when N>162 and when the mean age difference is less than 4 years. The assumptions of the ComBat model regarding the normality of residual distributions are not violated as the model becomes unstable.

**Conclusion:**

Prior to harmonization of DTI data with ComBat, the input cohort should be examined for size and covariate distributions of each site. Direct assessment of residual distributions is less informative on stability than bootstrap analysis. We caution use ComBat of in situations that do not conform to the above thresholds.

## 1 Introduction

Diffusion-weighted magnetic resonance imaging (dMRI) is a non-invasive imaging modality that provides insight into the white matter (WM) microstructure in the brain.^1^ In diffusion tensor imaging (DTI), the signal from a dMRI scan is modeled as tensors that describe the direction and degree of water diffusion at each voxel.^2^ One of the most common ways to study a DTI model is through scalar metrics. Two of the most common diffusion scalars are fractional anisotropy (FA), which describes the directedness of diffusion, and mean diffusivity (MD), which describes the average magnitude of diffusion. While DTI is limited in its ability to describe crossing axon fibers in the brain,^2^ it is still useful to study the changes in brain morphology due to disease or aging.^3^^–^^5^

Multi-site studies are desirable because they can increase sample size and incorporate population heterogeneity. However, multi-site studies can suffer from bias that is introduced by differences in data acquisition methods, study design, or other confounders that can affect the data.^6^ For MRI, factors, such as the brand of the scanner, the magnet strength, head coils used, acquisition protocols, and other imaging differences introduce site bias and uncertainty in the images.^7^ Diffusion imaging is especially sensitive to different acquisition parameters.^8^^–^^10^ Matsui et al. showed that even after preprocessing to correct for scanning distortions and artifacts common to diffusion imaging, the inter-site variability is still significant.^9^

To perform multi-site studies, site bias must be removed in a process called harmonization. For image level harmonization, where site bias is removed at the voxel level, a common technique is harmonization of the rotationally invariant spherical harmonic (RISH) features derived from images.^11^ Several deep learning algorithms have also been proposed for harmonization.^12^^,^^13^ One of the most common methods for DTI harmonization is ComBat, a statistical approach, where site bias is removed from features extracted from the images. Originally designed to remove batch effects in the field of genomics,^14^ ComBat has been adapted for image-level harmonization of DTI data to remove site biases.^15^ Other studies have used ComBat for harmonization of diffusion scalar metrics,^16^ and several different extensions of ComBat have been proposed as well that are covered in a recent review.^17^

There have been multiple previous studies assessing the reliability of ComBat for harmonizing multi-site medical imaging data. Zindler et al. assessed ComBat’s inflation of false positive results in the context of different sample sizes and number of features harmonized for genomic data.^18^ Bell et al. evaluated ComBat for harmonization of magnetic resonance spectroscopy (MRS) data.^19^ Cabini et al. examined ComBat harmonization of radiomic features extracted from CT images of lung cancer patients.^20^ Richter et al. validated ComBat and variations on a travelling cohort in terms of ability to remove site bias without removing true biological effect from structural and diffusion MRI.^21^ Orlhac et al. examined deeper than only harmonization potential and assessed different situations and use cases for ComBat in the context of harmonizing image-derived biomarkers from PET scans.^22^ Parekh et al. posited sample size requirements under different Mahalanobis distances between datasets for structural MRI features, with larger distances corresponding to greater site biases.^23^ A recent review of image harmonization listed the use cases of ComBat.^24^ Yet, these studies did not investigate the boundaries for which ComBat harmonization can still reliably estimate site bias in the context of dMRI. We seek to establish statistically based suggestive guidelines for situations in which ComBat can be used to harmonize data extracted from DTI.

## 2 Methods

We create a “silver standard” cohort from two datasets with DTI and match participants according to demographic covariates. We bootstrap subsets from this cohort and run ComBat harmonization on the subsets, with each bootstrap defined by the cohort parameters of total sample size, imbalance of sample sizes between sites, and mean age difference between sites. We regress on the harmonized data to find trends of FA versus age. Additionally, we obtain the final shift and scale parameters estimated by ComBat from the data. We compare the estimates of regression coefficients and ComBat parameters obtained from the silver standard cohort to those obtained from the bootstrapped subsets to assess reliability of ComBat.

### 2.1 Data Acquisition

We consider two datasets containing both DTI and T1-weighted images: the Baltimore Longitudinal Study of Aging (BLSA)^25^^,^^26^ and the Vanderbilt Memory and Aging Project (VMAP).^27^ VMAP data were collected by Vanderbilt Memory and Alzheimer’s Center Investigators at Vanderbilt University Medical Center. BLSA DTI scans were acquired on a 3T Phillips scanner in 32 directions at a b-value of $700\;s/{\mathrm{mm}}^{2}$ with voxel dimensions of $2.2\times 2.2\times 2.2\;{\mathrm{mm}}^{3}$ that were resampled to $0.8125\times 0.8125\times 2.2\;{\mathrm{mm}}^{3}$. All VMAP DTI scans considered were acquired on a 3T Phillips scanner with an 8ch SENSE head coil in 32 directions at a b-value of $1000\;s/{\mathrm{mm}}^{2}$ with voxel dimensions of $2\times 2\times 2\;{\mathrm{mm}}^{3}$. For both sites, T1 scans were acquired in the same scanning session as the DTI images. BLSA T1 scans were acquired with voxel dimensions of $1.2\times 1\times 1\;{\mathrm{mm}}^{3}$ and VMAP T1 scans were acquired with voxel dimensions of $1\times 1\times 1\;{\mathrm{mm}}^{3}$.

### 2.2 Silver Standard Cohort

We consider both cognitively unimpaired and mild cognitive impairment (MCI) participants across VMAP and BLSA, matched by cognitive status, sex, and age within 4 years while keeping track of APOE2 positivity, APOE4 positivity, race/ethnicity, and years of education. The final size of our silver standard cohort is N=358, with each site contributing an equal number of participants (Table 1).

**Table 1:** Demographic information for each dataset in the silver standard cohort.

Measure	BLSA (n=159)	VMAP (n=159)
Mean age (yrs)	73.9	74.0
Age range (min, max)	(59.8, 91.9)	(60.0, 92.0)
Sex (% male)	59	59
Percentage cognitively healthy	94	94
Race (% non-Hispanic white)	84	94
APOE2 (% positive)	18	16
APOE4 (% positive)	28	29
Average education (yrs)	17.0	16.3

### 2.3 Data Processing and Pre-processing

**Fig. 1:**
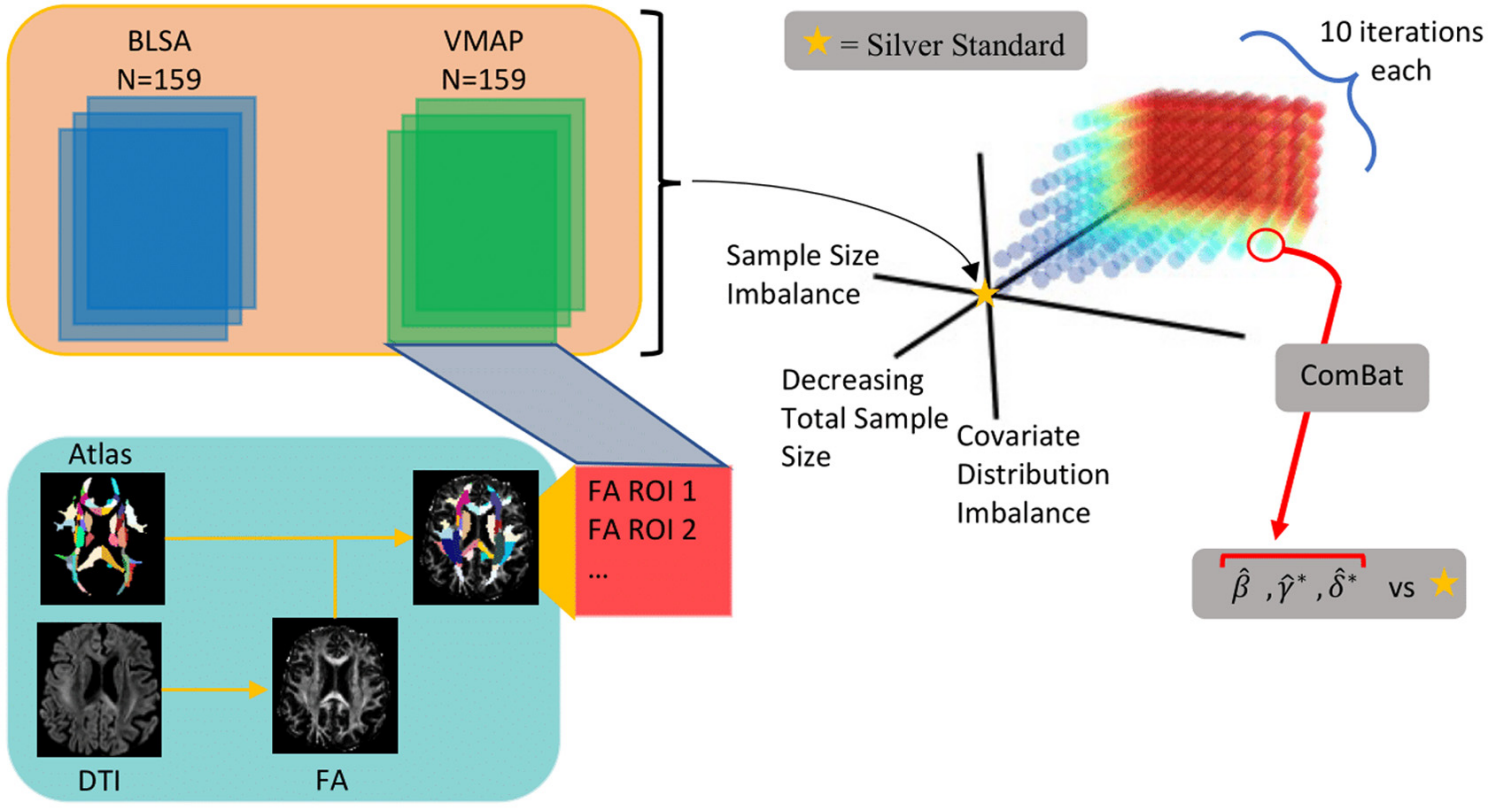
After registration of the JHU EVE-III Atlas, mean FA values were calculated in all the regions for each participant in the silver standard cohort. A point in the experimental space is “feasible” if the sample size for either site is at least N=6, the imbalance level does not result in N for either site exceeding the available number of participants for that site, and if sampling of participants yielded a covariate shift within 1 year of the target age difference between sites. For each feasible point in the experimental space, 10 bootstraps were subsampled from the silver standard cohort, and the FA values for the subsamples were harmonized by ComBat. The resulting parameters were then compared to those from the silver standard to determine reliability of ComBat at that location in the experimental space.

All DTI scans are preprocessed using v 1.0.8 of the PreQual preprocessing pipeline^28^ for denoising and to remove susceptibility-induced and eddy current distortions (Fig. 1). Fractional anisotropy (FA) and MD are calculated from the preprocessed data. The EVE Type-I, EVE Type-II, and EVE Type-III JHU atlases^29^^,^^30^ are registered to the diffusion space of each participant using ANTs SyN registration^31^ and FSL’s epi_reg.^32^ The T1 brain mask used for epi_reg is calculated via SLANT.^33^ The epi_reg transform is converted to the same format used by ANTs via the Convert3D^34^ tool developed by the ITK-SNAP team. The two transformations are then applied in a single registration step. After registration of the atlases to the diffusion space, mean FA, MD, AD, and RD are calculated for each region of the three atlases using the MRtrix3 software.^35^ We use the regions of interest (ROIs) from the EVE Type-III atlas, as they are all WM regions. The code used for the process is available at: (https://github.com/MASILab/AtlasToDiffusionReg).

### 2.4 ComBat Harmonization

The ComBat model proposed by Johnson et al.^14^ assumes that $Y_{isf}$, the original input scalars for feature f extracted from participant scan i that comes from site s are defined as

$$Y_{isf}=\alpha_{f}+X\beta_{f}+\gamma_{sf}+\delta_{sf}\varepsilon_{isf},$$ (1)

where $\alpha_{f}$ is the overall value for feature f; $\beta_{f}$ is the vector of regression coefficients that correspond to covariates in the covariate matrix, X; $\gamma_{sf}$ is the additive site bias shift for feature f at site s; $\delta_{sf}$ is the multiplicative site bias for feature f at site s; and $\varepsilon_{isf}$ is an error term that is assumed to be normally distributed with mean 0 and variance $\sigma_{f}^{2}$. ComBat requires three types of inputs: (i) the scalar values of the features that require harmonization; (ii) covariates, such as age or sex, that preserve the variability of the input data; and (iii) a site covariate that indicates variability to be removed. First, ComBat standardizes the distributions of the features to have similar means and variances

Zisf=Yisf−α^f−Xβ^fσ^f, (2)

where $Z_{isf}$ is the standardized data, $Y_{isf}$ is the unharmonized data, α^f is the estimator of feature f, β^f is the vector estimator of regression coefficients corresponding to X for feature f, and σ^f is the estimated standard deviation of feature f calculated as

σ^f2=1N∑is(Yisf−α^f−Xβ^f−γ^sf)2, (3)

where N is the total number of samples. It is necessary to constrain

∑snsγ^sf=0, (4)

for identifiability, where $n_{s}$ is the number of samples coming from site s and $N=\sum_{s}n_{s}$. In other words, without the constraint in Eq. (4), the assumed model in Eq. (1) would have an infinite number of solutions. ComBat also assumes that the standardized data are normally distributed according to $Z_{isf}∼(\gamma_{sf},\delta_{sf}^{2})$. Then, for each site and feature combination, ComBat uses an empirical Bayes method to iteratively update the estimates of the shift, γ^sf, and scale, ${\hat{\delta }}_{sf}$, parameters via an expectation maximization (EM) algorithm. Upon convergence, the final parameter estimates, ${\hat{\gamma }}_{sf}^{*}$ and δ^sf*, are used to create the batch-adjusted values $Y_{isf}$ using the equation:

Y^isf=σ^f(Yisf−α^f−Xβ^f−γ^sf*δ^sf*)+α^f+Xβ^f. (5)

We use (i) the mean FA values for 112 of the 118 regions of the EVE Type-III atlas for each participant; (ii) covariates of age, sex, cognitive status, race, education, APOE2 carrier status, and APOE4 carrier status, the covariates used in DTI harmonization by Yang et al.;^36^ and (iii) a covariate indicating the dataset the participant scan came from. Six regions are excluded from consideration because the registration process resulted in regions with zero volume, which could induce a large shift in the mean of the distribution of mean FA values for the cohort. Such shifts could have substantial impact on the ComBat harmonization procedure. The education covariate is a continuous variable indicating years of education, and the APOE2 and APOE4 covariates are categorical variables indicating the presence or absence of the respective APOE allele. The cognitive status covariate indicates whether the participant is cognitively unimpaired or is diagnosed with MCI. The version of ComBat used for this analysis is implemented by Fortin et al. (https://github.com/Jfortin1/neuroCombat).^15^

### 2.5 Experimental Search Space

Bayer et al. highlighted a variety of factors that might influence the performance of ComBat.^17^ To examine the robustness of ComBat, we use 740 different permutations of 19 levels of total sample size, 10 levels of sample size imbalance, and 6 levels of covariate shift (Fig. 1). Total sample size is the number of participants whose features are input to ComBat for harmonization. In the context of this analysis, we define sample size imbalance to be the ratio X:10 of participants from one site relative to the other site, where 10:10 is perfect balance of sample size between sites. We consider levels from X=1 to X=10. To compare different experimental permutations, we keep VMAP as the single site whose sample size changes with respect to BLSA. For the covariate shift, the value is the difference in the mean age of the participants from a single dataset compared to the other one. Fortin et al. demonstrated ComBat harmonization of datasets with different age ranges, but we evaluate performance at multiple levels to provide a more controlled and comprehensive analysis of changes in performance.^37^ For all levels of difference, we consider scenarios both when the mean age for VMAP is greater than BLSA and when the mean age of VMAP is less.

**Fig. 2:**
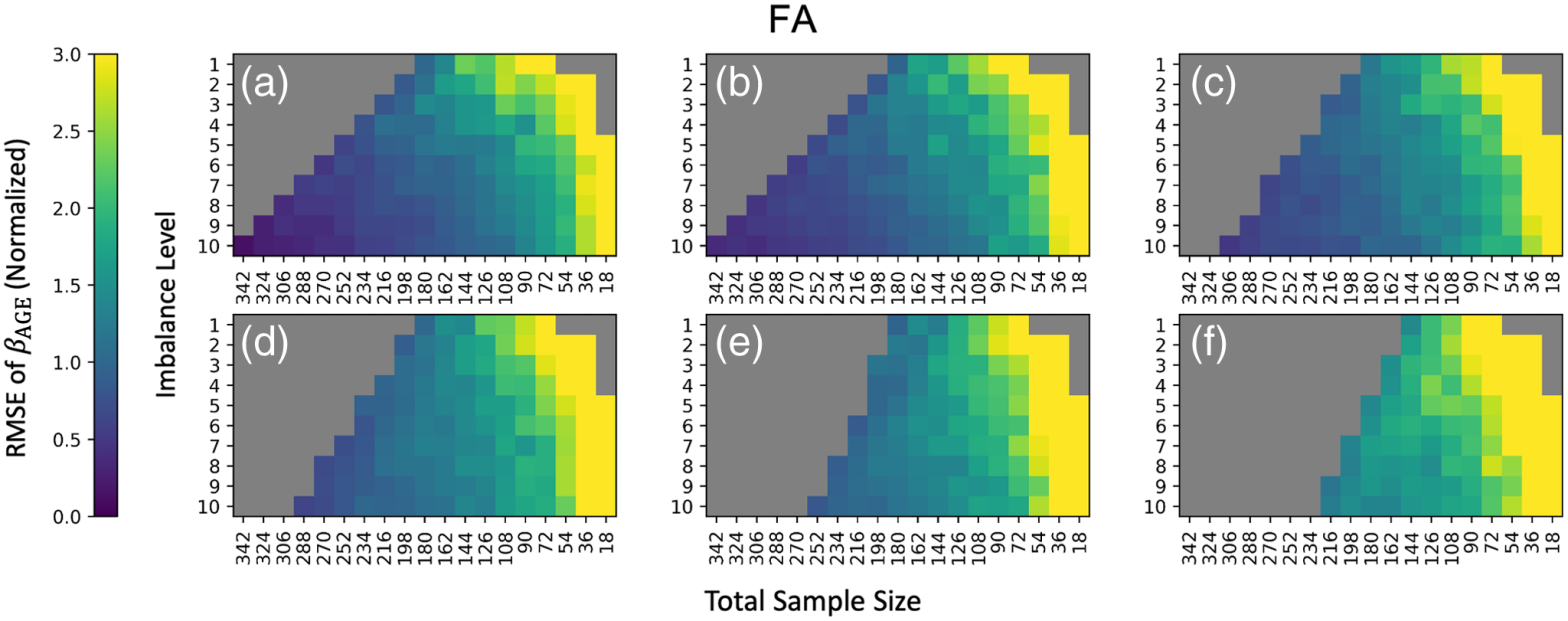
The root mean squared error (RMSE) of standardized $\beta_{\mathrm{AGE}}$ estimates for mean FA versus age compared to the silver standard indicate that ComBat is not stable with all experimental permutations considered, as the error increases when the cohort changes to have an average mean age difference between VMAP and BLSA of (a) 0 years, (b) 2 years, (c) 4 years, (d) 6 years, (e) 8 years, and (f) 10 years. The values represent the mean normalized RMSE across EVE Type-III Atlas regions averaged across 10 iterations of each feasible point in the experimental space. For each subplot, total sample size of the cohort is on the x-axis and sample size imbalance is on the y-axis, where Y:10 represents Y participants at VMAP for every 10 at BLSA. Any non-feasible experimental permutations are represented in gray.

### 2.6 Bootstrapping Experimental Space

We bootstrap 10 simulations at each of these experimental permutations through sampling from the silver standard cohort without replacement. For each iteration, ComBat harmonization is performed as described in Sec. 2.4 to obtain the harmonized data and estimated parameters γ^sf* and δ^sf* for each region and site combination. The mean FA vs Age regressions are calculated according to Sec. 2.7. Bootstrapping subsamples of the silver standard can be done either with or without replacement. As sampling with replacement can introduce artifacts in the data, such as repeated participants, we choose to sample without replacement. As a consequence, not all permutations in the experimental space defined in Sec. 2.5 are feasible. In addition, the subsamples at some permutations will be more highly correlated than others due to lack of participant variability for a site that fit the experimental criteria. We also do not include any permutations that result in any site having fewer than six participants.

### 2.7 Mean FA Versus Age Regression

To estimate the associations of FA with age from the harmonized data, we perform a linear regression

Y^ROI∼1+XAGEβAGE+XSEXβSEX+XSITEβSITE+XMCIβMCI+ε, (6)

where Y^ROI is the ComBat harmonized mean FA for an ROI in the EVE Type-III atlas. Covariates of race, education, APOE2 status, and APOE4 status are not used for the linear regression, as they do not significantly impact FA values in the harmonized cohort data. As FA has been shown to be negatively correlated with age,^3^ we use $\beta_{\mathrm{AGE}}$ to examine the changes among the experimental permutations. According to the central limit theorem (CLT), as N goes to infinity, the distribution of the means will tend to $N∼(\mu ,\frac{\sigma^{2}}{N})$.^38^ Thus, we expect the standard deviation of these means to be inversely proportional to $\sqrt{N}$ as N approaches infinity. Applying the CLT to our bootstrap analysis, we expect the mean squared error of $\beta_{\mathrm{AGE}}$ to tend to be $(X_{\mathrm{AGE}}^{T}X_{\mathrm{AGE}}{)}^{-1}\sigma^{2}$, so its root mean squared error would be inversely proportional to $\sqrt{N}$.

### 2.8 Comparison to Silver Standard

To evaluate the robustness of ComBat at each experimental permutation, we compare to the silver standard cohort using three different error metrics: (i) the average root mean square error in normalized effect size for $\beta_{\mathrm{AGE}}$ across all regions, (ii) the average root mean squared error of γ^sf* across all regions, and (iii) the average root mean squared error of the log of δ^sf* across all regions. The standard error for each regression estimate is also obtained from the linear regression estimation. We normalize $\beta_{\mathrm{AGE}}$ for each region by dividing by its respective silver standard standard error value from the regression estimation to compare $\beta_{\mathrm{AGE}}$ effect size across all regions for the permutations. Unlike $\beta_{\mathrm{AGE}}$, we cannot get silver standard estimates for the standard errors of γ^sf* and δ^sf*, so we cannot normalize the values with reference to the silver standard. Additionally, since γ^sf* and δ^sf* are estimated iteratively via the EM algorithm, they are highly dependent on each other, and normalizing them independently may introduce bias. Thus, we leave γ^sf* and δ^sf* unchanged from ComBat for comparisons to the silver standard. We also look at the average root mean squared errors of the differences between ${\hat{\gamma }}_{\mathrm{BLSA},f}^{*}$ and γ^VMAP,f* and between the log of ${\hat{\delta }}_{\mathrm{BLSA},f}^{*}$ and the log of ${\hat{\delta }}_{\mathrm{VMAP},f}^{*}$ across all regions in order to assess the relative scalings and shifts of the feature distributions.

### 2.9 Checking Assumptions

To determine if the instability of ComBat is related to the assumptions that the model makes, we assess the following.

1. Normality of residuals: from Eq. (1), ComBat assumes that the error/noise in the features being harmonized is normally distributed about the regression line fit by the model with a mean of zero and some variance $\sigma_{f}^{2}$.
2. Distributions of scaling and shifting: for the parametric version of ComBat, ${\hat{\gamma }}_{sf}^{*}$ for each site s is normally distributed, while ${\hat{\delta }}_{sf}^{*}$ for each site follows an inverse gamma distribution.
3. Covariates for ComBat are uncorrelated.

Note that the ComBat model for the data in Eq. (1) represents biological covariates as causing linearly independent variation in the data. Further, ComBat assumes that the biological variation in the data is separable from the variation due to site/scanner biases, which requires the biological covariates to not be strongly correlated with the batch variable. We assess (1) and (2) by evaluating the negative log likelihoods of the residual distributions compared to the assumed prior distributions. For the residuals of the model and ${\hat{\gamma }}_{sf}^{*}$, we use the mean and standard deviations of the empirical distributions to generate a normal distribution fit to the data and assess the average negative log likelihood across all empirical data points compared to the generated normal distribution. For ${\hat{\delta }}_{sf}^{*}$, we estimate the inverse gamma distribution as

$$\text{Inv Gamma}(x)=\frac{{\overset{˜}{\beta }}^{\overset{˜}{\alpha }}}{\Gamma (\overset{˜}{\alpha })}(1/x{)}^{\overset{˜}{\alpha }+1}\;\exp(-\overset{˜}{\beta }/x),$$ (7)

where Γ is the gamma function and $\tilde{\alpha },\tilde{\beta }$ are the shape and scaling parameters of the distribution respectively that we calculate as

$$\overset{˜}{\alpha }=\frac{\mu_{IG}^{2}}{\sigma_{IG}^{2}+2},$$ (8)

$$\tilde{\beta }=\mu (\tilde{\alpha }-1),$$ (9)

where $\mu_{IG}$ is the mean and $\sigma_{IG}^{2}$ is the variance of ${\hat{\delta }}_{sf}^{*}$. We calculate the average negative log likelihood across all ComBat estimated values compared to the generated inverse gamma distribution. For the residuals, ${\hat{\gamma }}_{sf}^{*}$, and ${\hat{\delta }}_{sf}^{*}$, we compare the average negative log likelihood values to the respective silver standard values to assess whether the experimental runs violate the assumptions more or less than the silver standard cohort. We also assess normality of the experimental residuals with the Anderson–Darling test.

### 2.10 Comparison to Other Linear Models

To assess the stability of ComBat compared to other linear models, we also perform the same analysis of root mean squared error in normalized $\beta_{\mathrm{AGE}}$ compared to the silver standard on the same data subsets of ComBat experimental runs for an ordinary least square (OLS) and linear mixed effects (LME) model. The OLS is the same as Eq. (6), but without the prior ComBat harmonization of the data. The LME is modeled as

Y^ROI∼1+XAGEβAGE+XSEXβSEX+XSITEbSITE+XMCIβMCI+ε, (10)

where $b_{\mathrm{SITE}}$ is a random effects term for the site covariate.

## 3 Results

**Fig. 3:**
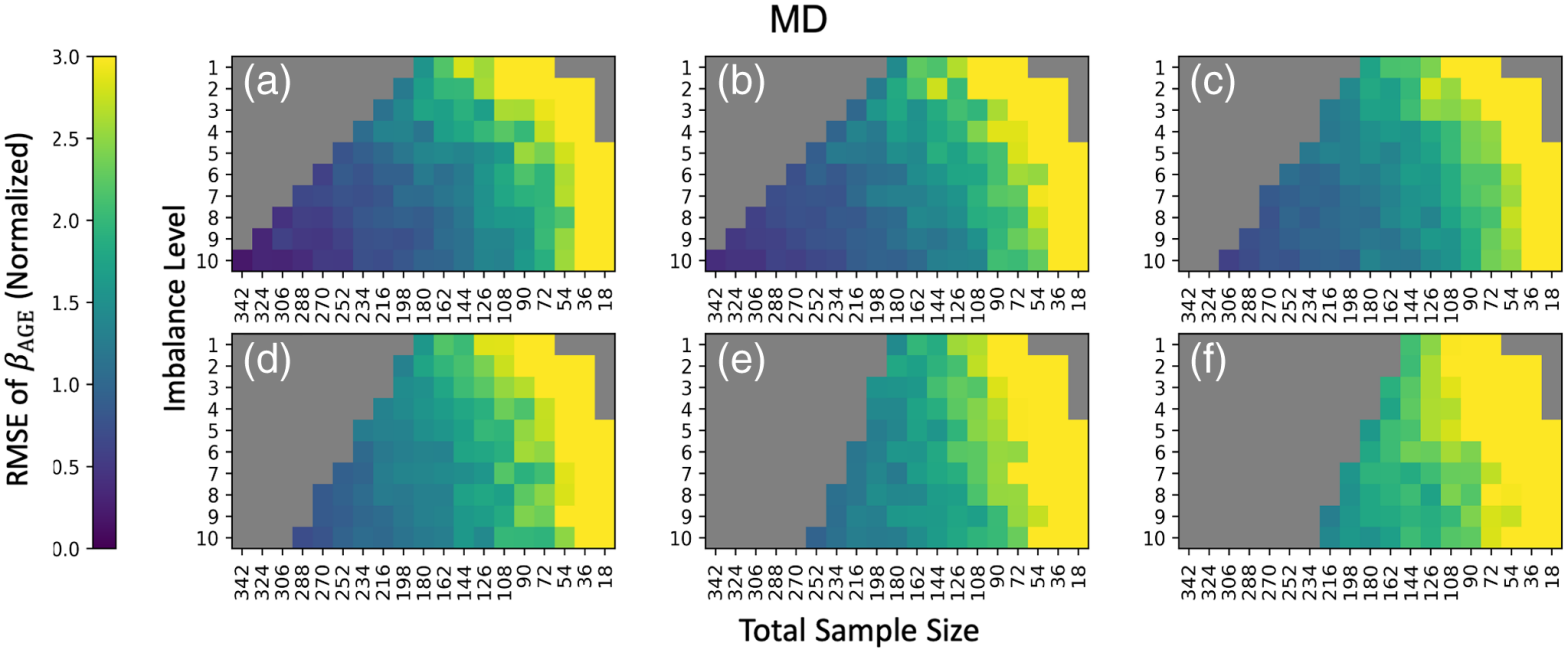
Comparing to Fig. 2, the RMSE of standardized $\beta_{\mathrm{AGE}}$ estimates for MD vs age show instability in ComBat as well, since the error also increases when the cohort changes to have an average mean age difference between VMAP and BLSA of (a) 0 years, (b) 2 years, (c) 4 years, (d) 6 years, (e) 8 years, and (f) 10 years. Data are plotted in the same manner as Fig. 3.

To make a comprehensive comparison of experimental parameters, we visualize the three-dimensional feasibility matrix as 2D slices along the covariate shift axis with total sample size and sample size imbalance as the x and y axes (Figs. 2 and 3). Additionally, since there are 112 ROIs to consider, we condense information regarding $\beta_{\mathrm{AGE}}$ for mean FA and mean MD to a single scalar by averaging the standardized effect size change of $\beta_{\mathrm{AGE}}$ across all ROIs. The corresponding results using all covariates input to ComBat rather than just those specified in Eq. (6) can be found in Fig. S1 in Supplementary Material 1. We say that ComBat performs well if the average standardized effect size change across ROIs is closer to zero. The general trend in performance for ComBat is a decrease in performance as we move further away from the silver standard with our experimental parameters. This decrease in performance is evidenced by the gradation in color from purple to yellow. Experimental parameters that are not feasible are grayed out. To assess if the ComBat residuals are larger at one site compared to the other, we visualize the site-wise residuals for mean FA and mean MD (Fig. S2 in Supplementary Material 1). We observe that both sites are represented evenly across the entire residual distribution. Estimates of $\beta_{\mathrm{AGE}}$, ${\hat{\gamma }}_{sf}^{*}$, δ^sf* and the log of δ^sf*, and the standard error of $\beta_{\mathrm{AGE}}$ for the silver standard can be found in Supplementary Material 2 as a CSV file.

### 3.1 Total Sample Size and $\beta_{\mathrm{AGE}}$

**Fig. 4:**
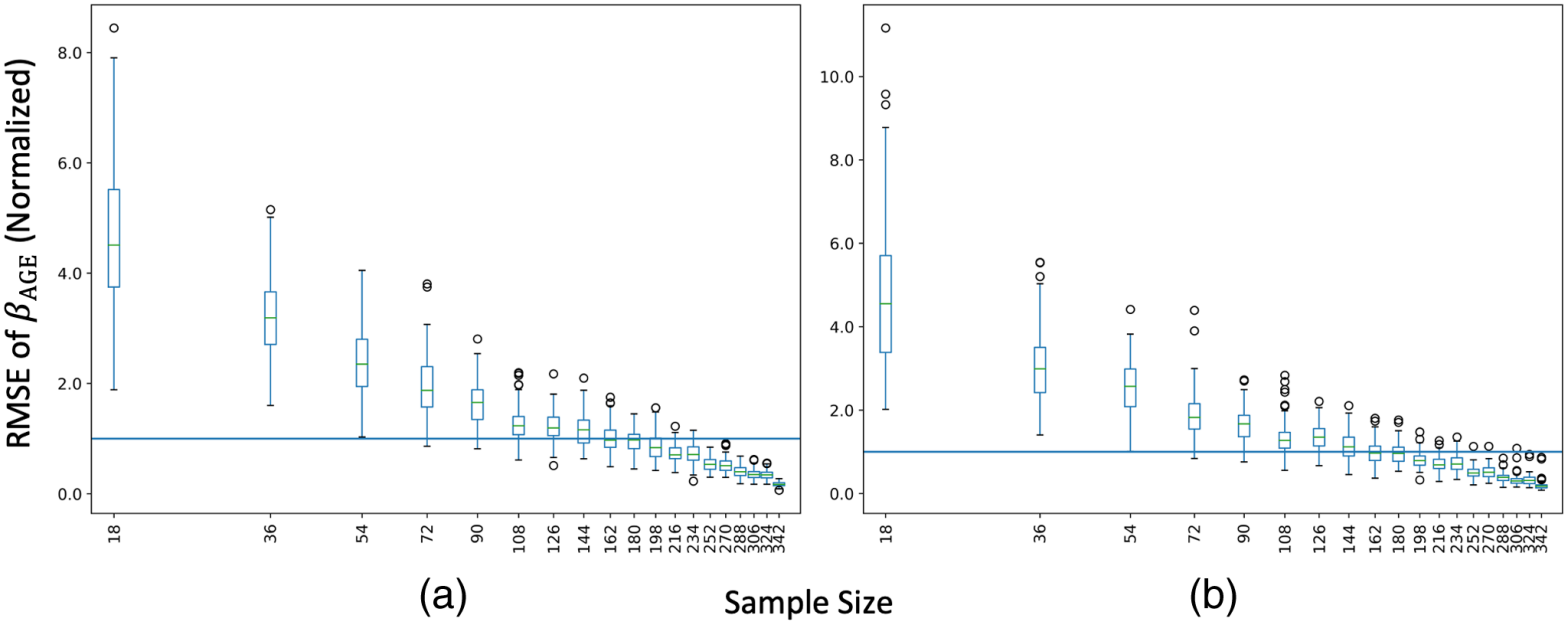
(a) For decreasing sample size, the expected trend for a well-behaved model is error increasing by a factor of $\sqrt{N}$. Thus, the trend in error in the logarithmic space is linear with increasing N. We consider ComBat to be “stable” with respect to N when the RMSE for standardized $\beta_{\mathrm{AGE}}$ of mean FA versus age compared to the silver standard is below 1 (blue line) or the error follows the error trend stated. On this criterion, we suggest that ComBat is unstable when N<162, as over 50% of the errors of ROIs for N=144 are above 1 and the increase in error in not linear in the logarithmic space. (b) RMSE for standardized $\beta_{\mathrm{AGE}}$ of mean MD versus age shows ComBat remaining stable for N>162, indicating that different DTI scalars have different levels of sensitivity to changes in sample size for ComBat. To observe the effect of only sample size on ComBat, only permutations with an imbalance level of 10:10 and no covariate shift were considered.

To evaluate ComBat’s reliability with respect to N, we plot root mean squared error of normalized $\beta_{\mathrm{AGE}}$ for each ROI averaged across all 10 iterations against the total sample size (Fig. 4). The total sample size is spaced logarithmically, so the expected trend in error as N increases will be a linear decrease with respect to logarithmic increases in sample size. At small sample sizes, the decrease in error does not follow this trend. We only consider experimental permutations that have a zero-year covariate shift and a 10:10 imbalance in sample size. We consider ComBat to be “stable” as long as $\beta_{\mathrm{AGE}}$ for 50% of the ROIs are within one standard deviation of the respective silver standard, and “unstable” otherwise. For harmonization of mean FA values, we observe that ComBat becomes unstable at sample sizes of N<162. For a more conservative threshold, where all ROIs are within one standard deviation away, we observe instability for N<252.

### 3.2 Sample Size Imbalance and $\beta_{\mathrm{AGE}}$

**Fig. 5:**
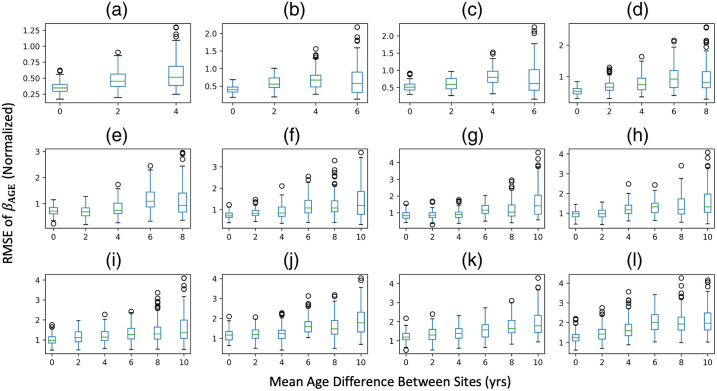
RMSE for standardized $\beta_{\mathrm{AGE}}$ of mean FA versus age compared to the silver standard for sample sizes of (a) N=306, (b) N=288, (c) N=270, (d) N=252, (e) N=234, (f) N=216, (g) N=198, (h) N=180, (i) N=162, (j) N=144, (k) N=126, and (l) N=108. Covariate shift does not seem to have a definitive threshold at which the error in estimation of $\beta_{\mathrm{AGE}}$ is much larger compared to the respective experimental run with no covariate shift. For an all-encompassing threshold ambiguous to the size of N, we suggest a maximum covariate shift of 2 years between sites because a covariate shift of either 4 or 6 years increases the error in estimation of $\beta_{\mathrm{AGE}}$ depending on N. Only experimental permutations that have an imbalance ratio of 10:10 were considered.

To examine the error in $\beta_{\mathrm{AGE}}$ estimation with respect to sample size imbalance, we plot the root mean squared error of $\beta_{\mathrm{AGE}}$ for each ROI averaged across all 10 iterations against the sample size imbalance for both sample sizes that would make ComBat stable and unstable (Fig. 5, Fig. S3 in Supplementary Material 1). We only consider experimental permutations that have a zero-year covariate shift. We do not observe sample size imbalance to have an effect on the estimation of $\beta_{\mathrm{AGE}}$, as the error fluctuates for all levels of imbalance.

### 3.3 Covariate Shift and $\beta_{\mathrm{AGE}}$

**Fig. 6:**
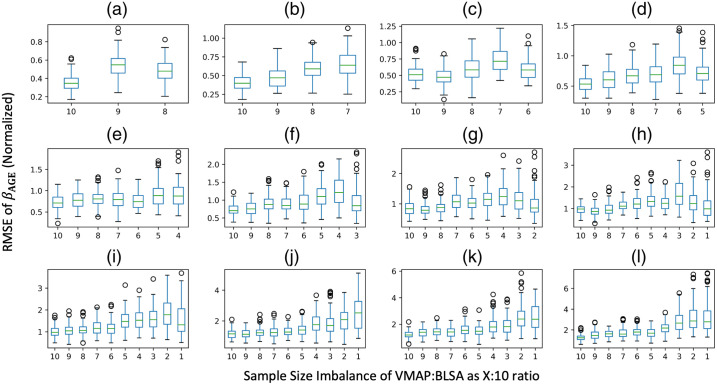
Sample size imbalance alone does not substantially affect estimation of $\beta_{\mathrm{AGE}}$ for mean FA harmonization; only at smaller N does it appear to have an effect. However, this is likely due to the small sample size at these experimental permutations. Only experimental permutations that have a covariate shift of 0 years were considered. Panels (a)–(l) are the same N as Fig. 5.

To examine the error in $\beta_{\mathrm{AGE}}$ estimation with respect to covariate shift, we plot the root mean squared error of $\beta_{\mathrm{AGE}}$ for each ROI averaged across all 10 iterations against the covariate shift (Fig. 6, Fig. S4 in Supplementary Material 1). We only consider experimental permutations that have a 10:10 sample size imbalance level. For stable sample sizes, we observe stability along the covariate shift axis at mean age differences of up to 2 to 4 years between sites. As this threshold fluctuates among sample sizes, we consider a conservative threshold at 2 years and a looser threshold at 4 years. The average effect size for each level of covariate shift can be found in Table S1 in Supplementary Material 1.

### 3.4 Comparison to Silver Standard - γ^sf* and ${\hat{\delta }}_{sf}^{*}$

**Fig. 7:**
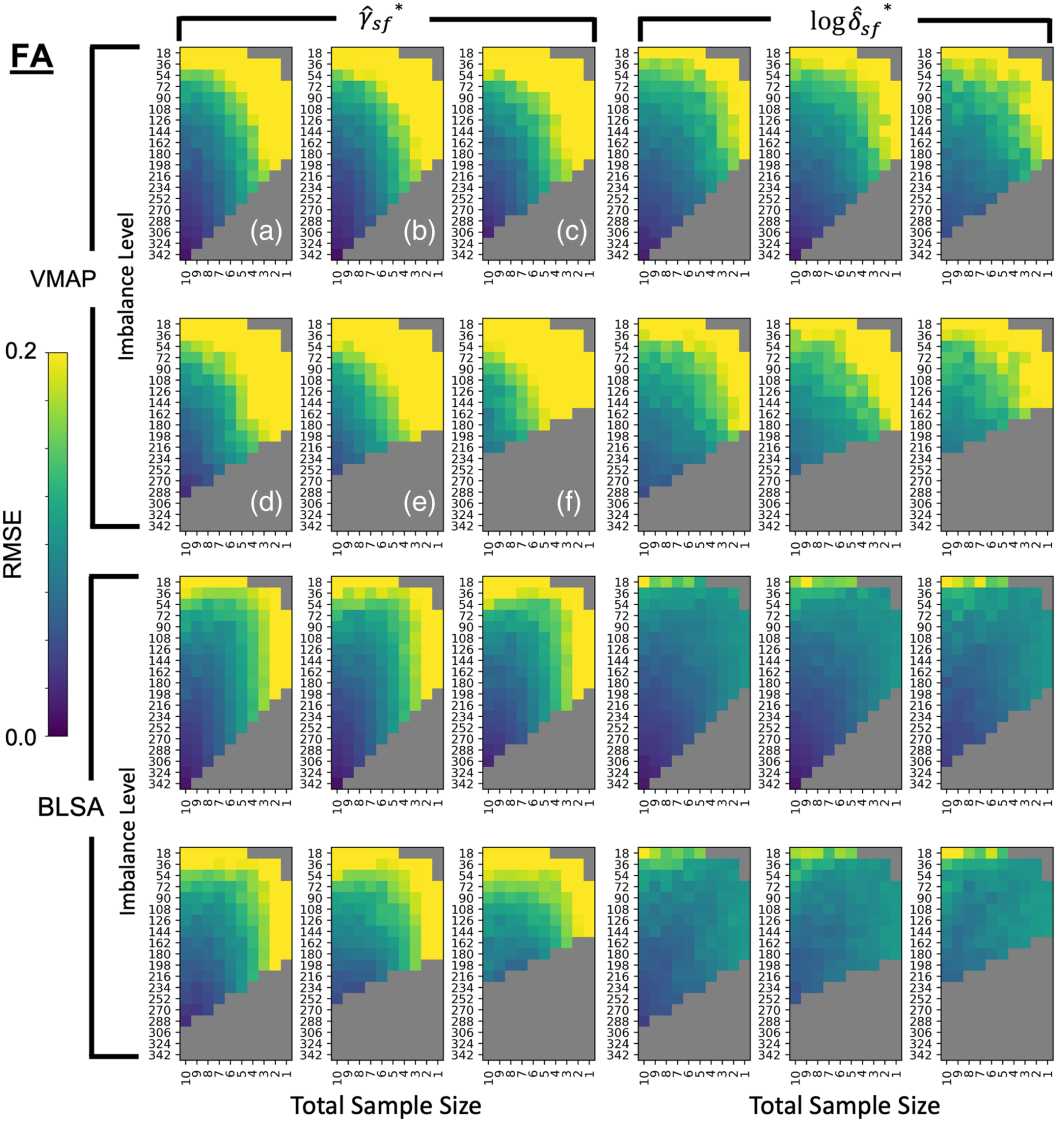
γ^sf* and log δ^sf* do not follow the same trend in error as $\beta_{\mathrm{AGE}}$ for harmonization of mean FA values. (Top left) RMSE error in ${\hat{\gamma }}_{sf}^{*}$ estimates for VMAP averaged across ROIs with total sample size on the y-axis and sample size imbalance on the x-axis at covariate shift levels of (a) 0 years, (b) 2 years, (c) 4 years, (d) 6 years, (e) 8 years, and (f) 10 years. (Top right) Error in log δ^sf* estimates for VMAP averaged across ROIs and plotted in the same order as top left. Bottom left and bottom right plots are the RMSE in ${\hat{\gamma }}_{sf}^{*}$ and log δ^sf* estimates for BLSA respectively, with slices along the covariate shift axis presented the same as top left. For ComBat to accurately estimate site bias, ${\hat{\gamma }}_{sf}^{*}$ and ${\hat{\delta }}_{sf}^{*}$ should be as close to the silver standard values as possible. Thus, we suggest a maximum covariate shift of 2 years, an imbalance of 9:10 and a total sample size of N>252 for stability in both ComBat parameters, as these experimental parameters have a relative error close to zero.

**Fig. 8:**
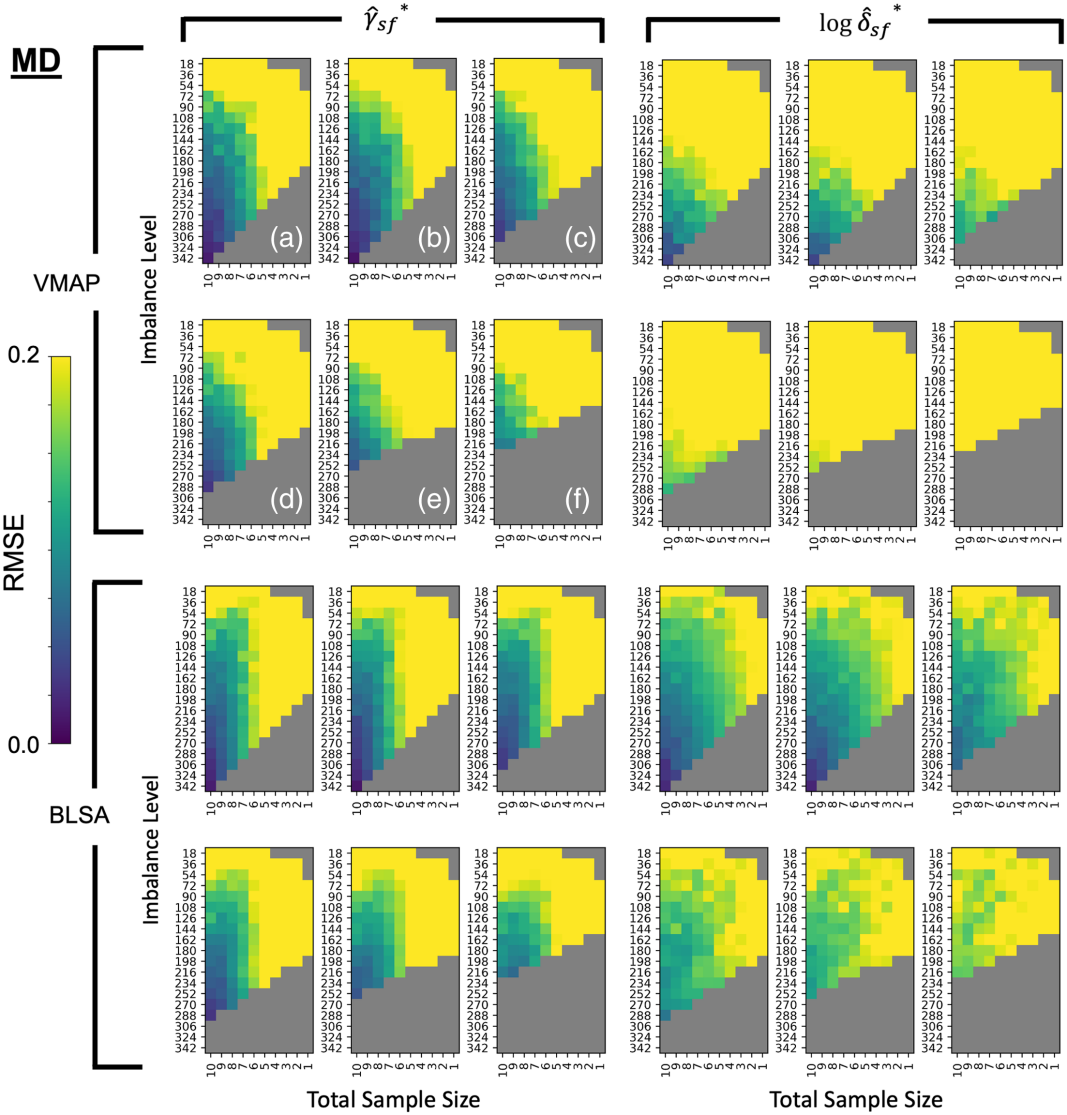
RMSE error in estimates for (top left) VMAP γ^sf*, (top right) VMAP log δ^sf*, (bottom left) BLSA ${\hat{\gamma }}_{sf}^{*}$, and (bottom right) BLSA log δ^sf* for harmonization of mean MD values increase much more quickly than they do for harmonization of FA values. Slices along the covariate shift axis are plotted similarly to Fig. 7.

To examine the error in harmonization, we perform a comprehensive visualization of root mean squared error of γ^sf* and the log of ${\hat{\delta }}_{sf}^{*}$ for each site at each feasible experimental parameter (Figs. 7 and 8). Unlike sample size with $\beta_{\mathrm{AGE}}$, we do not have expectations of well-behaved models for changes in γ^sf* and ${\hat{\delta }}_{sf}^{*}$. As the γ^sf* and ${\hat{\delta }}_{sf}^{*}$ estimates are the measures of site bias, we consider ComBat to be unstable with any deviation from the silver standard values, as any error would result either in site bias not being removed completely or removing variability attributed to biological factors. The gradation in color indicates increasing error as we move further away from the silver standard.

For γ^sf*, in terms of sample size, we observe a threshold of around N≥252 for ComBat stability, similar to the conservative threshold for sample size in error estimation of $\beta_{\mathrm{AGE}}$; along the sample size imbalance axis, we observe a threshold of around a 10 : 9 imbalance ratio between sites; and along the covariate shift axis, we observe a threshold of around four years. For δ^sf*, in terms of sample size, we observe a threshold of around N≥308 for ComBat stability; along the sample size imbalance axis, any imbalance in sample size results in ComBat instability; and along the covariate shift axis, we observe a threshold of up to 2 years in mean age difference between sites for ComBat stability.

**Fig. 9:**
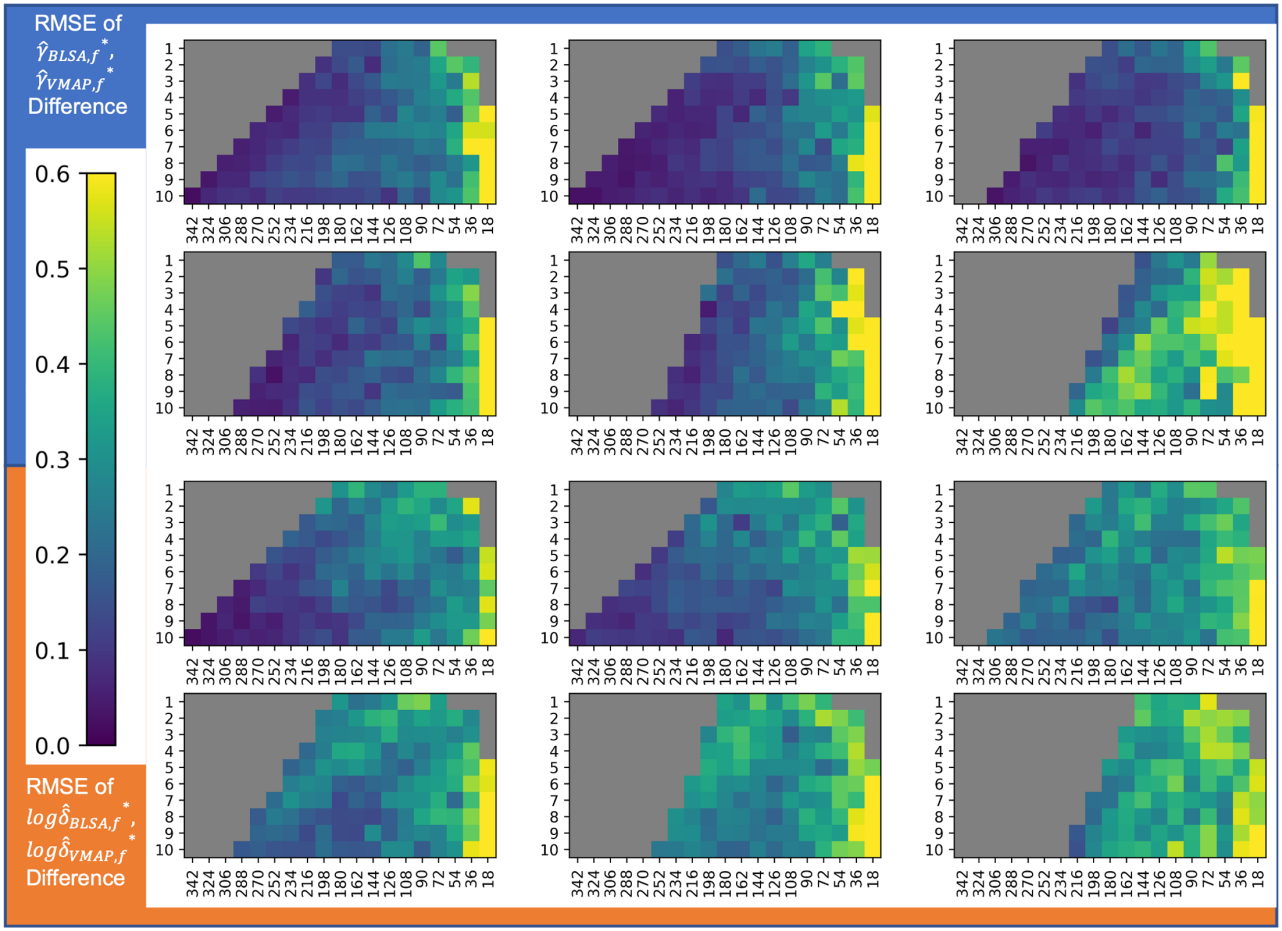
RMSE for γ^BLSA,f*−γ^VMAP,f* of experimental runs compared to the silver standard averaged across ROIs shows a similar threshold for stability of around N≥252 as the lone RMSE of γ^sf* for both sites. However, the stability of the difference for log δ^BLSA,f*−log δ^VMAP,f* shows a looser threshold of N≥252 compared to the lone RMSE of log δ^sf* for both sites for mean FA harmonization.

For the shift differences compared to those of the silver standard, we observe changes in γ^BLSA,f*−γ^VMAP,f* that are less consistently responsive to changes in the experimental parameters (Fig. 9, Fig. S5 in Supplementary Material 1) than for the site-wise root mean squared errors of γ^sf* for both sites. Still, we observe a similar threshold of around N≥252 for γ^sf* in terms of respective shift stability, a threshold of 9:10 for imbalance ratio between sites, and a covariate shift threshold of four years. For log(δ^BLSA,f*)−log(δ^VMAP,f*), we similarly observe less consistent responses to changes in the experimental parameters than for the site-wise root mean squared errors. Similar to the site-wise estimation errors, we observe that any imbalance results in ComBat instability and a threshold of 2 years for imbalance ratio and covariate shift, respectively. However, we observe a threshold of N≥252 for log(δ^sf*) in terms of respective scaling stability, which is less restrictive than that of the lone site-wise estimates.

### 3.5 Analysis of Assumptions

**Fig. 10:**
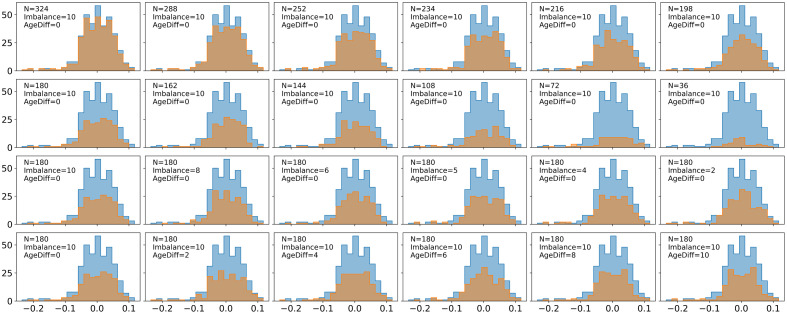
The residuals from the ComBat model for the silver standard (blue, N=358) do not adhere to the assumption of normality given the heavy left tail. As we step further from the silver standard in terms of sample size, imbalance, and covariate shift, we expect the residuals to become even less normally distributed if the assumption of residual normality directly impacts the error in $\beta_{\mathrm{AGE}}$ for experimental runs (blue). Decreasing sample size does not appear to consistently lessen the tail of the residual distributions. The residuals plotted above are for the left genu of the corpus callosum for mean FA harmonization.

**Fig. 11:**
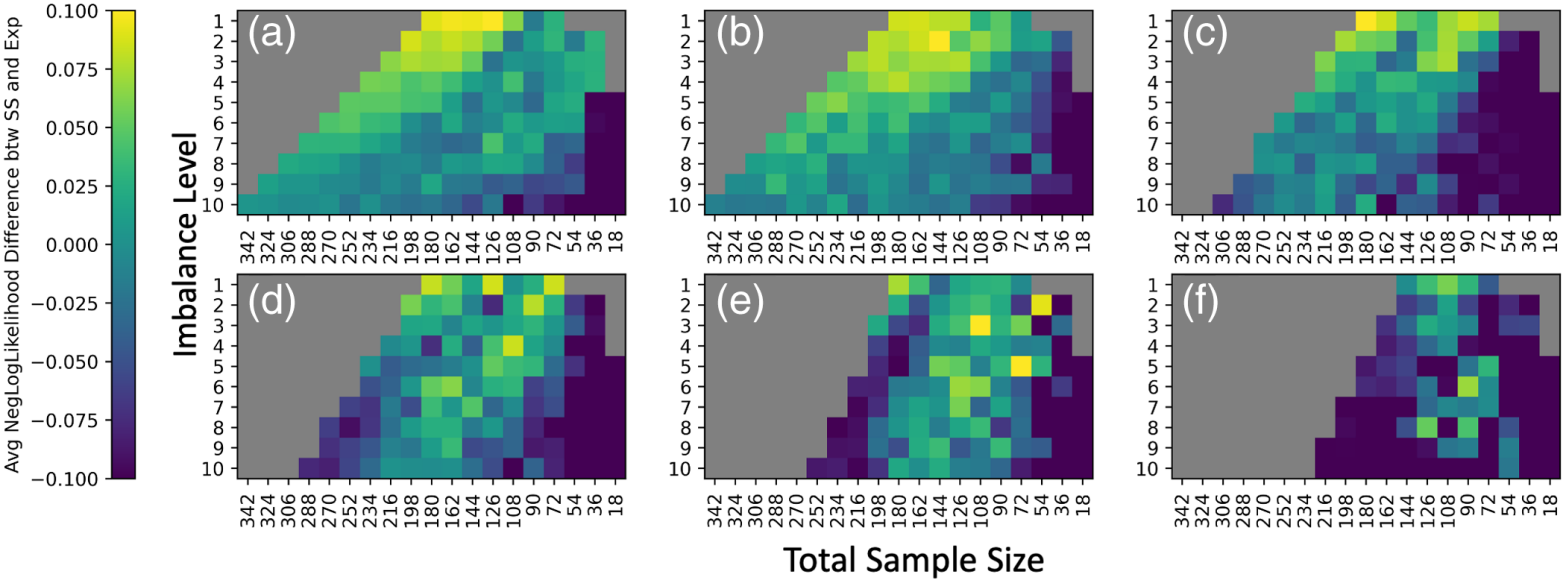
(a)–(f) The average negative log likelihood that the residual distribution follows a normal distribution (with mean and standard deviation estimated from the residual distribution) decreases as we decrease in sample size, and is smallest when the sample size is <50, suggesting that the residual distributions are more normal at low sample sizes for mean FA harmonization. This contrasts with the increasing error of $\beta_{\mathrm{AGE}}$ with decreasing sample size, suggesting that looking at the distribution of the assumptions alone cannot indicate if ComBat is appropriate for removing site biases of the given input cohort. Thus, we suggest the bootstrapping methodology to determine reliability of ComBat for site bias removal. Difference of average negative log likelihoods for residual distributions between experimental runs and the silver standard negative log likelihoods (averaged across all ROIs). Slices along the covariate shift axis are plotted similarly to Fig. 2.

**Fig. 12:**
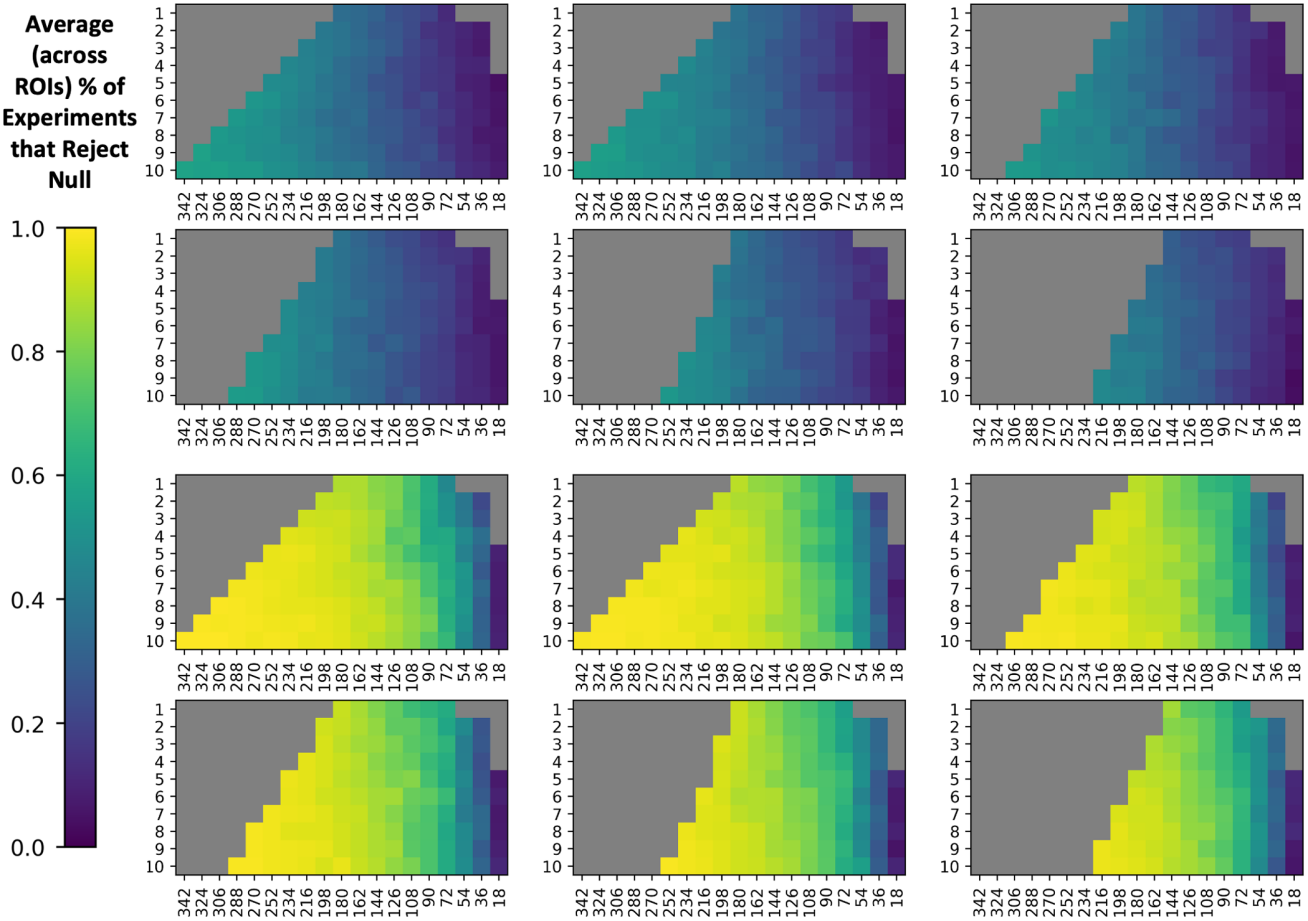
The Anderson–Darling test results suggest that the increase in error for $\beta_{\mathrm{AGE}}$, and thus the stability of ComBat, cannot be assessed by a decrease in normality of the residuals for either mean FA (top) or mean MD (bottom) harmonization.

We visualize distributions of experimental run residuals for mean FA compared to the silver standard residuals in Fig. 10 and plot the average of the negative log likelihoods of residuals in Fig. 11 (see Figs. S6 and S7 in Supplementary Material 1 as well). We do see an increase in the negative log likelihoods as we decrease sample size, in spite of an increase in error for the estimation of $\beta_{\mathrm{AGE}}$. At the smallest sample sizes, where the estimation for $\beta_{\mathrm{AGE}}$ has the most error, the negative log likelihood is at its most negative values, indicating the residuals for small sample sizes are, on average, more normally distributed than other experimental bootstraps and the silver standard. The results from the Anderson–Darling test are also in agreement with the negative log likelihood analysis for sample size, as the residuals are normally distributed more often at lower sample sizes, which do not follow the trend of increasing error of $\beta_{\mathrm{AGE}}$ with decreasing sample size (Fig. 12). We see a decrease in the negative log likelihoods as we move further from the silver standard along the covariate shift axis. We see a slight increase in the negative log likelihoods as we move further along the sample size imbalance axis, indicating that sample size imbalance may lead to violation of the assumptions of ComBat. However, the Anderson–Darling results suggest that neither imbalance ratio nor covariate shift have a consistent effect on the normality of the residuals.

**Fig. 13:**
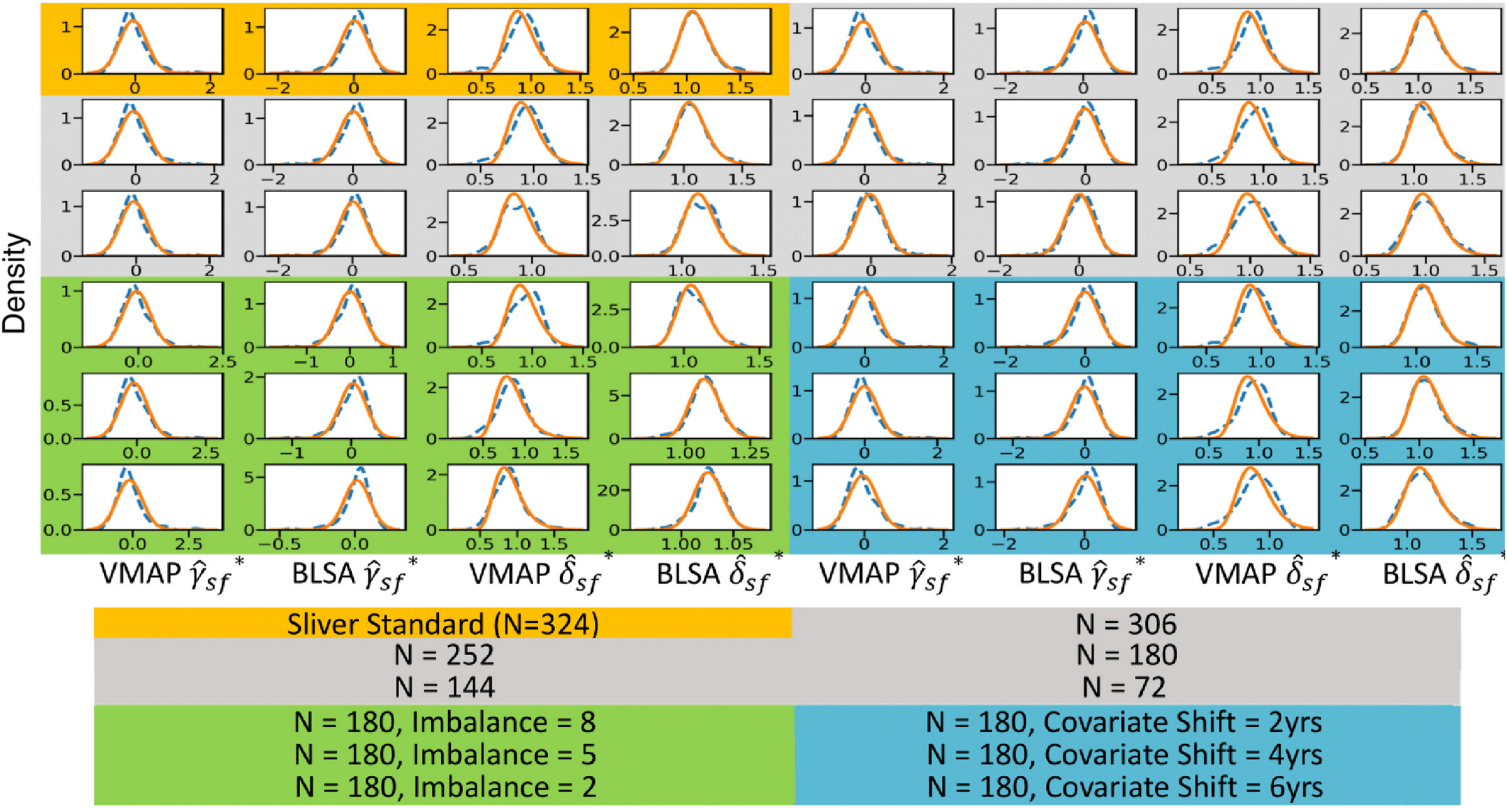
Despite changes in the experimental parameters compared to the silver standard, the empirical distributions (dotted blue) of the ${\hat{\gamma }}_{sf}^{*}$ and ${\hat{\delta }}_{sf}^{*}$ parameters for both sites in FA harmonization closely follow the respective estimated distributions. This adherence to the assumptions on ComBat does not correlate with the respective increasing error in estimates of $\beta_{\mathrm{AGE}}$ for experimental iterations that are further from the silver standard along the experimental axes. γ^sf* is assumed to have a normal distribution per site ${\hat{\delta }}_{sf}^{*}$ is assumed to have an inverse gamma distribution.

**Fig. 14:**
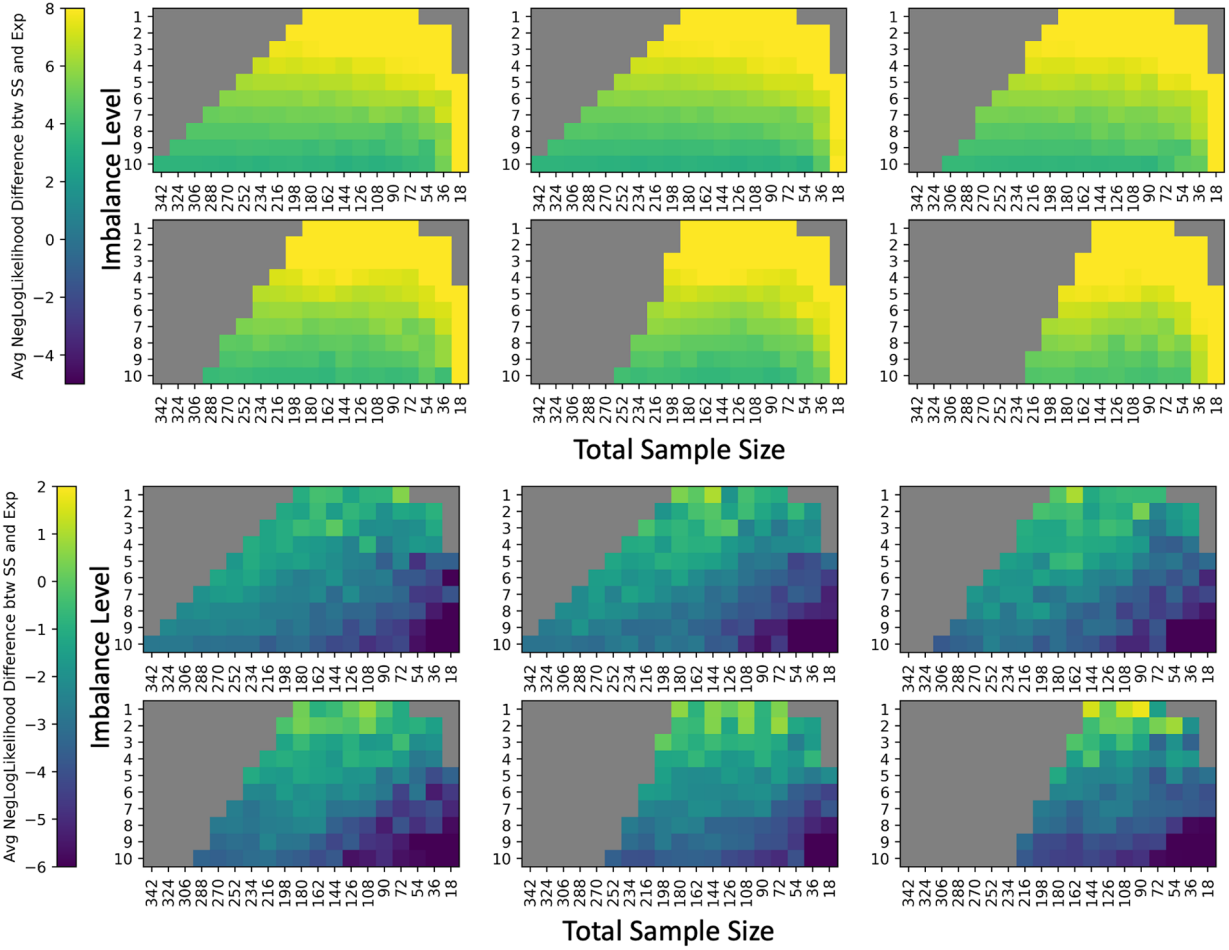
Similar to the ComBat model residuals, the VMAP average negative log likelihoods of the ${\hat{\gamma }}_{sf}^{*}$ estimates (top) for a normal distribution and ${\hat{\delta }}_{sf}^{*}$ estimates (bottom) for an inverse gamma distribution of experimental runs compared to the respective silver standard values do not correlate with the error trends for $\beta_{\mathrm{AGE}}$ in mean FA harmonization. This suggests that we cannot look at the distributions of ${\hat{\gamma }}_{sf}^{*}$ and ${\hat{\delta }}_{sf}$ to examine whether the input cohort is suitable for ComBat harmonization based on the premise of a violation of ComBat assumptions.

**Fig. 15:**
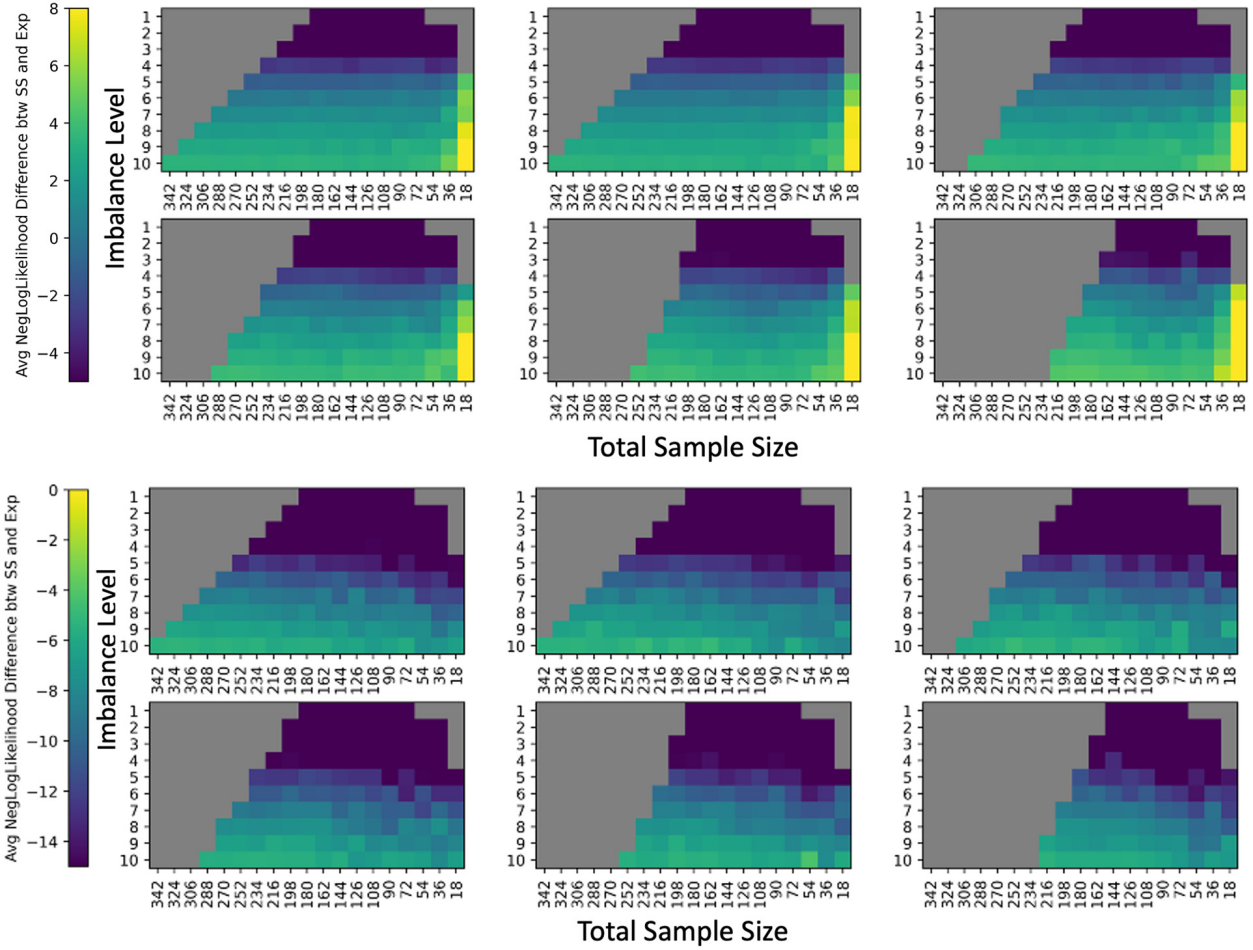
BLSA average negative log likelihoods for γ^sf* (top) and δ^sf* (bottom) compared to the respective silver standard values do not correlate with the error trends for $\beta_{\mathrm{AGE}}$ in mean FA harmonization.

**Fig. 16:**
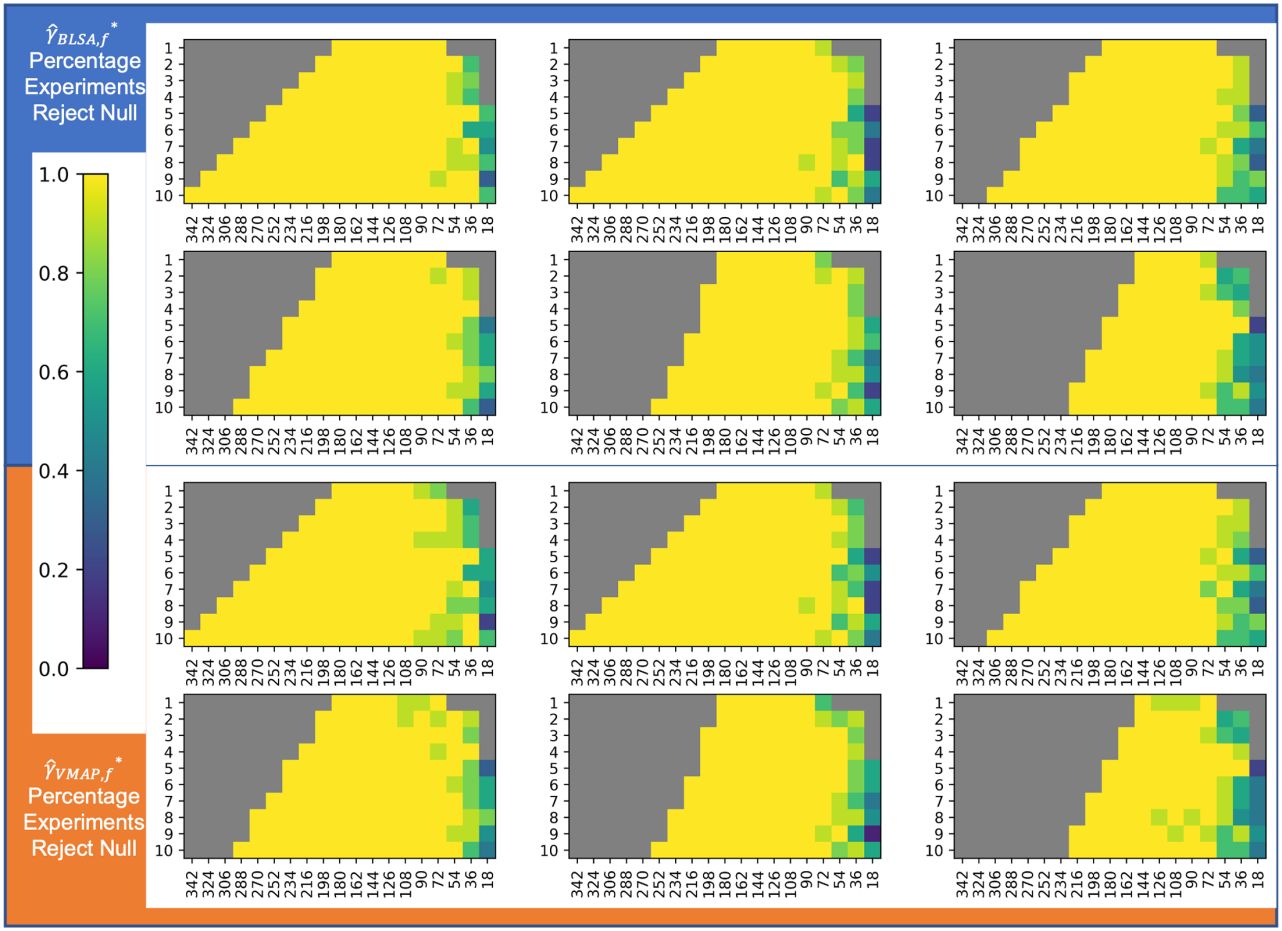
The Anderson–Darling test results suggest that the increase in error for $\beta_{\mathrm{AGE}}$, and thus the stability of ComBat, cannot be assessed by a decrease in normality of ${\hat{\gamma }}_{sf}^{*}$ for mean FA harmonization.

We visualize distributions of ${\hat{\gamma }}_{sf}^{*}$ and ${\hat{\delta }}_{sf}^{*}$ obtained from mean FA harmonization as kernel density estimates compared to respective prior distributions in Fig. 13 and plotted the average negative log likelihoods of both parameters in Figs. 14 and 15. Corresponding plots for mean MD harmonization can be found in Figs. S8–S10 in Supplementary Material 1. For ${\hat{\gamma }}_{sf}^{*}$, total sample size does not appear to have an effect on the shape of the distributions for experimental runs until N=36, at which point there is a large increase in the negative log likelihoods. For mean FA, we observe that ${\hat{\gamma }}_{sf}^{*}$ distributions are not normally distributed until around N=36 according to the Anderson–Darling test (Fig. 16), which agrees with the negative log likelihood values. The results from the Anderson–Darling test for mean MD harmonization are plotted in Fig. S11 in Supplementary Material 1. However, sample size imbalance appears to affect the distributions, as VMAP ${\hat{\gamma }}_{sf}^{*}$ deviate from normal and BLSA ${\hat{\gamma }}_{sf}^{*}$ tend to normal as we move further from the silver standard along the covariate shift axis. Covariate shift does not appear to affect the distribution of ${\hat{\gamma }}_{sf}^{*}$.

**Fig. 17:**
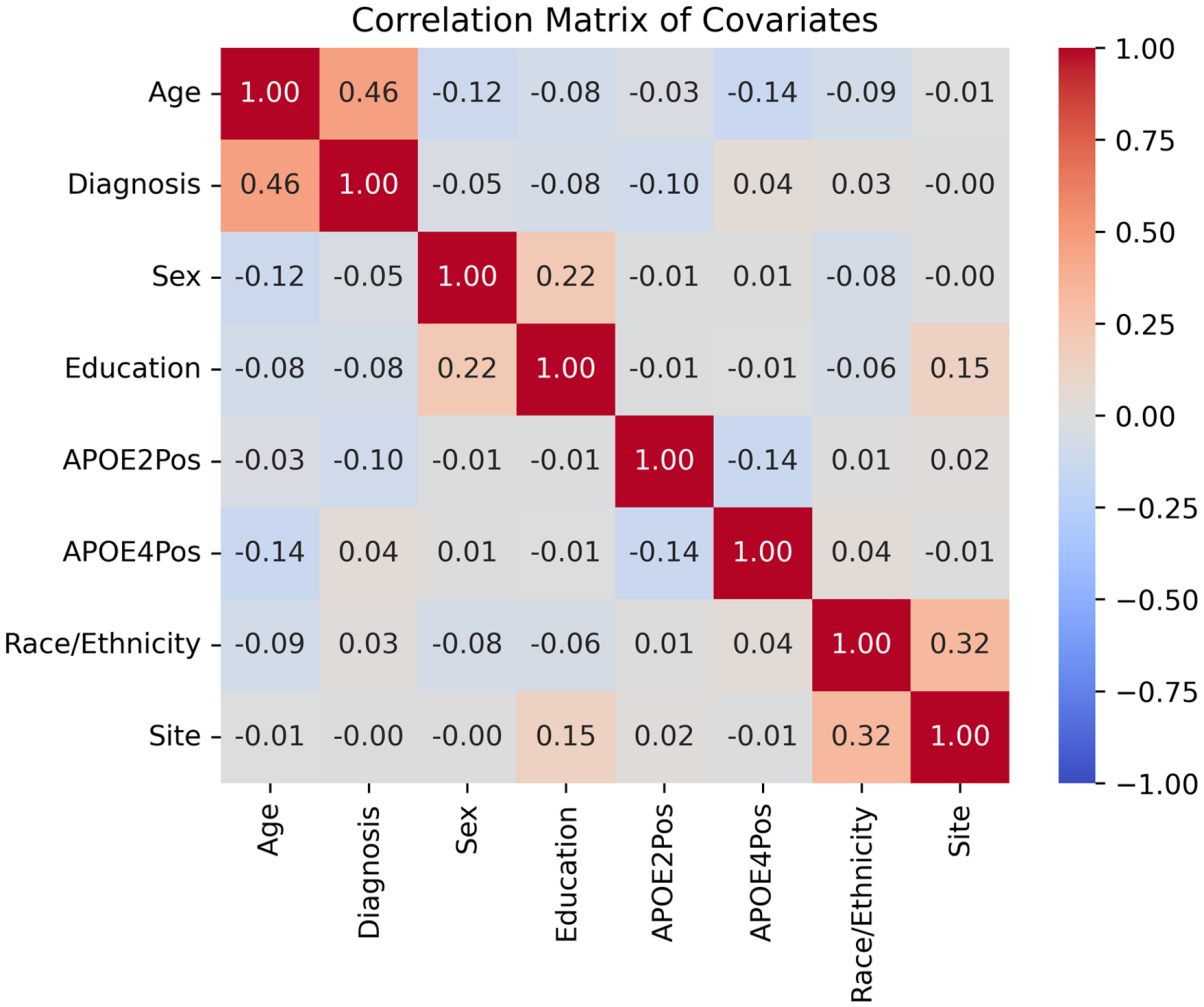
For the silver standard, the covariates are not highly correlated. We expect a correlation between age and diagnosis, as participants with MCI are more likely to be older. The correlations between race/ethnicity and site are non-zero but are substantially <1.

For δ^sf*, neither covariate shift nor sample size appears to affect the negative log likelihoods of the distributions. For VMAP, imbalance appears to have no effect on ${\hat{\delta }}_{sf}^{*}$, whereas a greater imbalance makes BLSA δ^sf* closer to an inverse gamma distribution, indicating that a larger proportion of the total sample size will make ${\hat{\delta }}_{sf}^{*}$ distributed more similarly to an inverse gamma distribution. We also calculate the correlation matrix of covariates for the silver standard cohort (Fig. 17) and do not find unexpectedly high correlations between any covariates.

### 3.6 Comparison to Other Linear Models

**Fig. 18:**
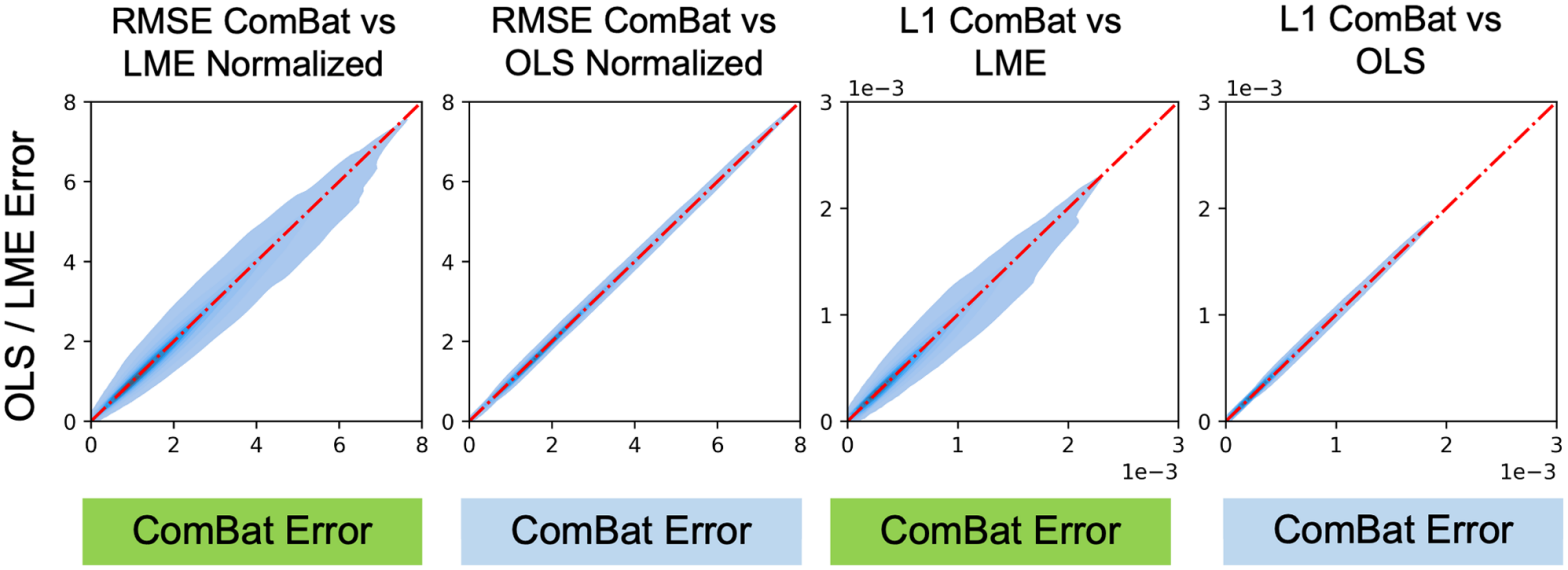
Pairwise comparisons for all experimental permutations of ComBat to OLS and LME models for $\beta_{\mathrm{AGE}}$ of mean FA suggest that ComBat is very similar to other linear models for pairwise DTI harmonization.

**Fig. 19:**
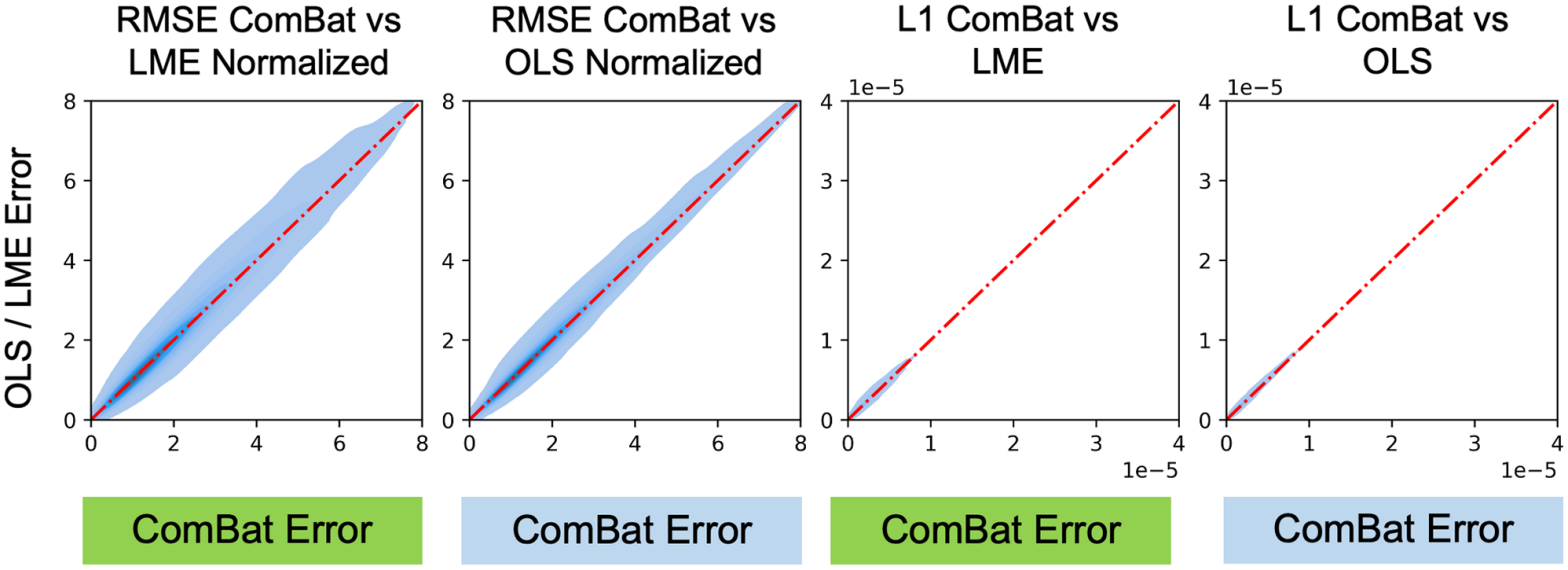
Pairwise comparisons for all experimental permutations of ComBat to OLS and LME models for $\beta_{\mathrm{AGE}}$ of mean MD suggest that ComBat is very similar to other linear models for pairwise DTI harmonization. For permutations with very large error, ComBat may have slightly more stability.

We do not observe asymmetry in the pairwise comparison of model errors for either mean FA or mean MD for ComBat versus OLS and LME (Figs. 18 and 19). Practically speaking, ComBat is similar to both LME and OLS when trying to harmonize DTI datasets with matched pairs.

## 4 Discussion

Orlhac et al. showed that for harmonization of PET imaging biomarkers between two sites, ComBat becomes less reliable at sample sizes less than N=20 to N=30 per site through a bootstrap analysis similar to ours.^22^ They also suggested in a latter conclusion that if covariates are used, N=20 to N=30 samples per covariate are used at each site. The work herein shows that their suggestion cannot be easily generalized to continuous covariates, such as age. Our results suggest that, for harmonization of dMRI measures, ComBat becomes unstable at even larger sample sizes per site. Given that DTI-derived data does not appear to be normally distributed, often having heavy tails, it is understandable that larger sample sizes are necessary in order for ComBat to remain stable in its estimates. For stability in estimates of ${\hat{\gamma }}_{sf}^{*}$ and δ^sf*, the differences between sites have looser thresholds than those for the lone site-wise estimates. We note that the assessment of the error in site-wise ${\hat{\gamma }}_{sf}^{*}$ and δ^sf* should be viewed with the lens of the identifiability issue [Eq. (4)], and assessments of the differences between sites for ${\hat{\gamma }}_{sf}^{*}$ and ${\hat{\delta }}_{sf}^{*}$ are more representative of the stability of ComBat estimates.

Johnson et al., the creators of the ComBat model, and Fortin et al., the first to harmonize dMRI data using ComBat, suggested at least N≈20 per site for ComBat harmonization to be reliable.^14^^,^^15^ Further, both posit that if the distributions of γ^sf* and δ^sf* follow the respective prior distributions, then parametric harmonization of ComBat is reliable. Our results suggest that the increase in estimation of $\beta_{\mathrm{AGE}}$ does not appear to correlate with a violation of the assumptions of ComBat, indicating that the reliability of ComBat cannot be assessed by plotting the distribution of the residuals, ${\hat{\gamma }}_{sf}^{*}$, and δ^sf*. This contradicts Johnson et al., Fortin et al., and Orlhac et al., suggesting that the ComBat model is more complex and nuanced than originally believed, especially in the context of dMRI data. Thus, to ensure a true evaluation of an input cohort to ComBat, we suggest a bootstrapping method similar to the one implemented in this work. Note, we do not suggest that ComBat or statistical harmonization are not important or reliable methods for harmonization of dMRI data. Rather, we advocate caution when assessing whether it is an appropriate model, particularly for harmonizing different cohorts with a disjoint covariate overlap or small sample sizes.

However, we note that not every multi-site dataset we wish to harmonize will be well matched like our silver standard and may thus require more data or stricter thresholds for stability of ComBat. Future work may wish to examine if the reliability of ComBat is similar for the same total sample sizes, but smaller sample sizes per site with more sites. Harmonization of three or more sites may also yield greater differences in error between the ComBat model and the other linear models. Additionally, we only consider three experimental axes along, which we could examine the reliability of ComBat. A deeper analysis could be performed using additional considerations listed in Bayer et al. that we do not address or other cohort modifications that could influence the performance of ComBat. We also only consider age for the covariate shift axis, whereas analysis of categorical covariate shifts or covariate shifts of multiple covariates could yield different results.

Another consideration that we do not examine in this work is the effect of image b-value correction prior to ComBat harmonization. For dMRI acquisitions with $500<b\text{-}\text{value}<1500\;s/{\mathrm{mm}}^{2}$, the diffusion-weighted signal is approximately linearly scalable in the logarithmic space, and previous work has shown that b-value correction using this approximation can help remove site differences.^39^^,^^40^ Given that the b-values for both VMAP and BLSA are different and fall within this range, future work could address if a prior b-value scaling would increase the stability of ComBat at smaller sample sizes or larger covariate shifts.

## Supplementary Material





## Data Availability

BLSA data utilized in this study were obtained from https://www.blsa.nih.gov/. Data are available from the authors upon request, and with permission from the Baltimore Longitudinal Study of Aging. VMAP data utilized in this study were obtained from https://www.vmacdata.org/vmap/data-requests. Data are available from the authors upon request and with permission from the Vanderbilt Memory and Alzheimer’s Center.

## References

[1] Alexander A. L. et al., “Diffusion tensor imaging of the brain,” Neurotherapeutics 4(3), 316–329 (2007). 10.1016/j.nurt.2007.05.011 17599699 PMC2041910

[2] O’Donnell L. J. Westin C.-F., “An introduction to diffusion tensor image analysis,” Neurosurg. Clin. N. Am. 22(2), 185–196 (2011). 10.1016/j.nec.2010.12.004 21435570 PMC3163395

[3] Sexton C. E. et al., “Accelerated changes in white matter microstructure during aging: a longitudinal diffusion tensor imaging study,” J. Neurosci. 34(46), 15425–15436 (2014). JNRSDS 0270-6474 10.1523/JNEUROSCI.0203-14.2014 25392509 PMC4228140

[r4] Shafer A. T. et al., “Accelerated decline in white matter microstructure in subsequently impaired older adults and its relationship with cognitive decline,” Brain Commun. 4(2), fcac051 (2022). 10.1093/braincomms/fcac051 35356033 PMC8963308

[5] Alm K. H. Bakker A., “Relationships between diffusion tensor imaging and cerebrospinal fluid metrics in early stages of the Alzheimer’s disease continuum,” J. Alzheimer’s Dis. 70(4), 965–981 (2019). 10.3233/JAD-181210 31306117 PMC6860011

[6] Jones D., Diffusion MRI: Theory, Methods, and Applications, Oxford University Press (2011).

[7] Panman J. L. et al., “Bias introduced by multiple head coils in MRI research: an 8 channel and 32 channel coil comparison,” Front. Neurosci. 13, 729 (2019).1662-453X 10.3389/fnins.2019.00729 31379483 PMC6648353

[8] Magnotta V. A. et al., “Multicenter reliability of diffusion tensor imaging,” Brain Connectivity 2(6), 345–355 (2012). 10.1089/brain.2012.0112 23075313 PMC3623569

[r9] Matsui J. T., “Development of image processing tools and procedures for analyzing multi-site longitudinal diffusion-weighted imaging studies,” Doctor of Philosophy, University of Iowa (2014).

[10] Zhu T. et al., “Evaluation of measurement uncertainties in human diffusion tensor imaging (DTI)-derived parameters and optimization of clinical DTI protocols with a wild bootstrap analysis,” J. Magn. Reson. Imaging 29(2), 422–435 (2009). 10.1002/jmri.21647 19161198

[11] Mirzaalian H. et al., “Harmonizing diffusion MRI data across multiple sites and scanners,” Lect. Notes Comput. Sci. 9349, 12–19 (2015). LNCSD9 0302-9743 10.1007/978-3-319-24553-9_2 PMC5045042 27754499

[12] Ning L. et al., “Cross-scanner and cross-protocol multi-shell diffusion MRI data harmonization: algorithms and results,” NeuroImage 221, 117128 (2020). NEIMEF 1053-8119 10.1016/j.neuroimage.2020.117128 32673745 PMC10243465

[13] Moyer D. et al., “Scanner invariant representations for diffusion MRI harmonization,” Magn. Reson. Med. 84(4), 2174–2189 (2020). MRMEEN 0740-3194 10.1002/mrm.28243 32250475 PMC7384065

[14] Johnson W. E. Li C. Rabinovic A., “Adjusting batch effects in microarray expression data using empirical Bayes methods,” Biostatistics 8(1), 118–127 (2007). 10.1093/biostatistics/kxj037 16632515

[15] Fortin J.-P. et al., “Harmonization of multi-site diffusion tensor imaging data,” NeuroImage 161, 149–170 (2017). NEIMEF 1053-8119 10.1016/j.neuroimage.2017.08.047 28826946 PMC5736019

[16] Zavaliangos-Petropulu A. et al., “Diffusion MRI indices and their relation to cognitive impairment in brain aging: the updated multi-protocol approach in ADNI3,” Front. Neuroinf. 13, 2 (2019). 10.3389/fninf.2019.00002 PMC6390411 30837858

[17] Bayer J. M. M. et al., “Site effects how-to and when: an overview of retrospective techniques to accommodate site effects in multi-site neuroimaging analyses,” Front. Neurol. 13, 923988 (2022). 10.3389/fneur.2022.923988 36388214 PMC9661923

[18] Zindler T. et al., “Simulating ComBat: how batch correction can lead to the systematic introduction of false positive results in DNA methylation microarray studies,” BMC Bioinf. 21(1), 271 (2020). BBMIC4 1471-2105 10.1186/s12859-020-03559-6 PMC7328269 32605541

[19] Bell T. K. et al., “Harmonization of multi-site MRS data with ComBat,” NeuroImage 257, 119330 (2022). NEIMEF 1053-8119 10.1016/j.neuroimage.2022.119330 35618196

[20] Cabini R. F. et al., “Preliminary report on harmonization of features extraction process using the ComBat tool in the multi-center ‘Blue Sky Radiomics’ study on stage III unresectable NSCLC,” Insights Imaging 13(1), 38 (2022). 10.1186/s13244-022-01171-1 35254525 PMC8901939

[21] Richter S. et al., “Validation of cross-sectional and longitudinal ComBat harmonization methods for magnetic resonance imaging data on a travelling subject cohort,” NeuroImage Rep. 2(4), 100136 (2022). 10.1016/j.ynirp.2022.100136 PMC9726680 36507071

[22] Orlhac F. et al., “A guide to ComBat harmonization of imaging biomarkers in multicenter studies,” J. Nucl. Med. 63(2), 172–179 (2022). JNMEAQ 0161-5505 10.2967/jnumed.121.262464 34531263 PMC8805779

[23] Parekh P. et al., “Sample size requirement for achieving multisite harmonization using structural brain MRI features,” NeuroImage 264, 119768 (2022). NEIMEF 1053-8119 10.1016/j.neuroimage.2022.119768 36435343 PMC7615107

[24] Hu F. et al., “Image harmonization: a review of statistical and deep learning methods for removing batch effects and evaluation metrics for effective harmonization,” Neuroimage 274, 120125 (2023). NEIMEF 1053-8119 10.1016/j.neuroimage.2023.120125 37084926 PMC10257347

[25] Shock N. W. Others A., Normal Human Aging: The Baltimore Longitudinal Study of Aging, Superintendent of Documents (1984).

[26] Ferrucci L., “The Baltimore Longitudinal Study of Aging (BLSA): a 50-year-long journey and plans for the future,” J. Gerontol. A Biol. Sci. Med. Sci. 63(12), 1416–1419 (2008). 10.1093/gerona/63.12.1416 19126858 PMC5004590

[27] Moore E. E. et al., “Increased left ventricular mass index is associated with compromised white matter microstructure among older adults,” J. Am. Heart Assoc. 7(13), e009041 (2018). 10.1161/JAHA.118.009041 29945917 PMC6064880

[28] Cai L. Y. et al., “PreQual: an automated pipeline for integrated preprocessing and quality assurance of diffusion weighted MRI images,” Magn. Reson. Med. 86(1), 456–470 (2021). MRMEEN 0740-3194 10.1002/mrm.28678 33533094 PMC8387107

[29] Oishi K. et al., “Atlas-based whole brain white matter analysis using large deformation diffeomorphic metric mapping: application to normal elderly and Alzheimer’s disease participants,” NeuroImage 46(2), 486–499 (2009). NEIMEF 1053-8119 10.1016/j.neuroimage.2009.01.002 19385016 PMC2885858

[30] Mori S. et al., “Stereotaxic white matter atlas based on diffusion tensor imaging in an ICBM template,” NeuroImage 40(2), 570–582 (2008). NEIMEF 1053-8119 10.1016/j.neuroimage.2007.12.035 18255316 PMC2478641

[31] Tustison N. J. et al., “Large-scale evaluation of ANTs and FreeSurfer cortical thickness measurements,” NeuroImage 99, 166–179 (2014). NEIMEF 1053-8119 10.1016/j.neuroimage.2014.05.044 24879923

[32] Jenkinson M. et al., “FSL,” NeuroImage 62(2), 782–790 (2012). NEIMEF 1053-8119 10.1016/j.neuroimage.2011.09.015 21979382

[33] Huo Y. et al., “3D whole brain segmentation using spatially localized atlas network tiles,” NeuroImage 194, 105–119 (2019). NEIMEF 1053-8119 10.1016/j.neuroimage.2019.03.041 30910724 PMC6536356

[34] Yushkevich P. A. et al., “User-guided 3D active contour segmentation of anatomical structures: significantly improved efficiency and reliability,” NeuroImage 31(3), 1116–1128 (2006). NEIMEF 1053-8119 10.1016/j.neuroimage.2006.01.015 16545965

[35] Tournier J.-D. et al., “MRtrix3: a fast, flexible and open software framework for medical image processing and visualisation,” NeuroImage 202, 116137 (2019). NEIMEF 1053-8119 10.1016/j.neuroimage.2019.116137 31473352

[36] Yang Y. et al., “White matter microstructural metrics are sensitively associated with clinical staging in Alzheimer’s disease,” Alzheimer’s Dementia Diagn. Assess. Dis. Monit. 15(2), e12425 (2023). 10.1002/dad2.12425 PMC10192723 37213219

[37] Fortin J.-P. et al., “Harmonization of cortical thickness measurements across scanners and sites,” NeuroImage 167, 104–120 (2018). NEIMEF 1053-8119 10.1016/j.neuroimage.2017.11.024 29155184 PMC5845848

[38] Kwak S. G. Kim J. H., “Central limit theorem: the cornerstone of modern statistics,” Kor. J. Anesthesiol. 70(2), 144–156 (2017). 10.4097/kjae.2017.70.2.144 PMC5370305 28367284

[39] Cetin Karayumak S. et al., “Retrospective harmonization of multi-site diffusion MRI data acquired with different acquisition parameters,” NeuroImage 184, 180–200 (2019). NEIMEF 1053-8119 10.1016/j.neuroimage.2018.08.073 30205206 PMC6230479

[40] Newlin N. R. et al., “MidRISH: unbiased harmonization of rotationally invariant harmonics of the diffusion signal,” bioRxiv 2023.08.12.553099 (2023). 10.1016/j.mri.2024.03.033 PMC11283839 38537892

