# Supplementary material for: Empirical assessment of the assumptions of ComBat with diffusion tensor imaging
Source: J Med Imaging (Bellingham). 2024 Apr 17;11(2):024011. doi: 10.1117/1.JMI.11.2.024011 (PMC11034156; doi:10.1117/1.JMI.11.2.024011)
Supplement: Supplementary file 1 [file JMI_011_024011_SD001.pdf]

Table S1. Average effect size (calculated as Cohen's D) for each level of age imbalance. We note that experiments with larger mean age differences are only at smaller sample sizes, so we expect the pooled standard deviation to be larger, which increases the effect size.

MeanAgeDiff	0	2	4	6	8	10
AvgEffectSize	0.082	0.297	0.626	1.005	1.429	1.929

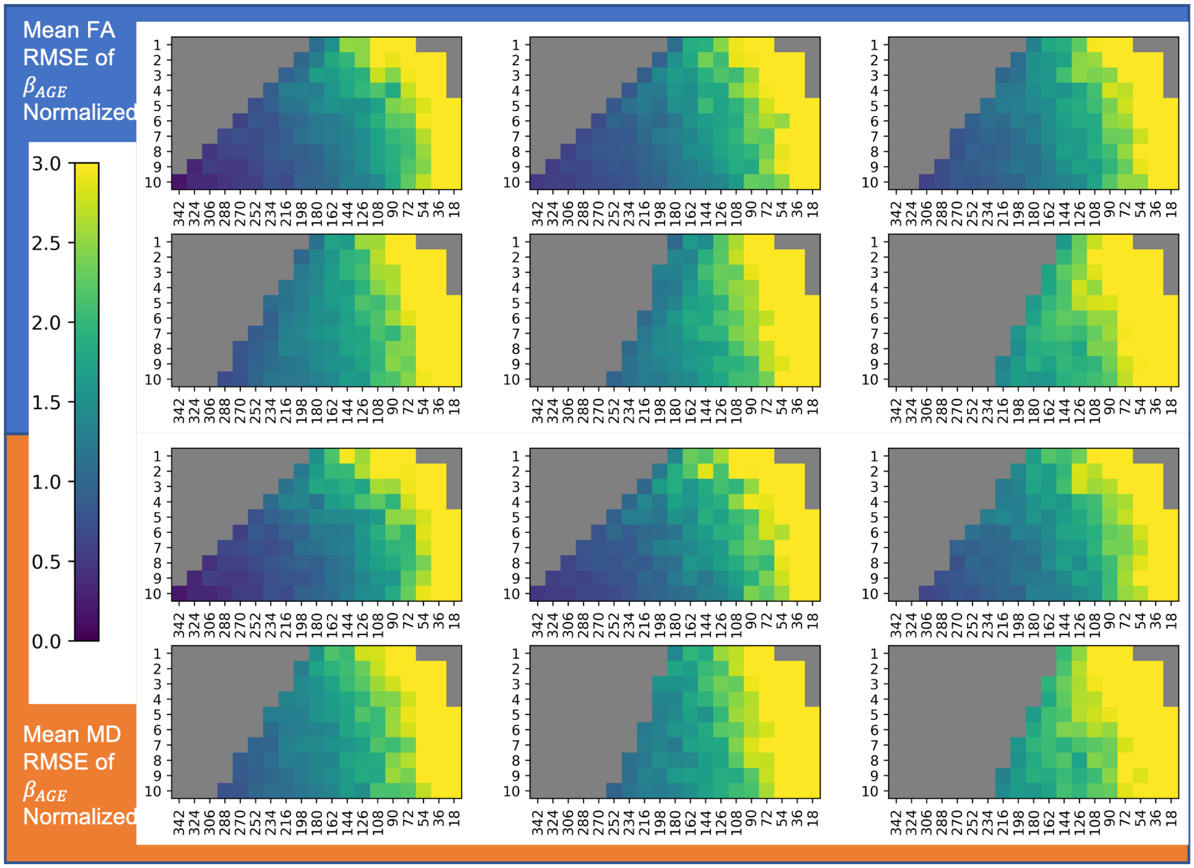

Figure S1. The error in β_{AGE} when using all covariates for the post-ComBat linear model is practically very similar when using the linear model from Eq. 6.

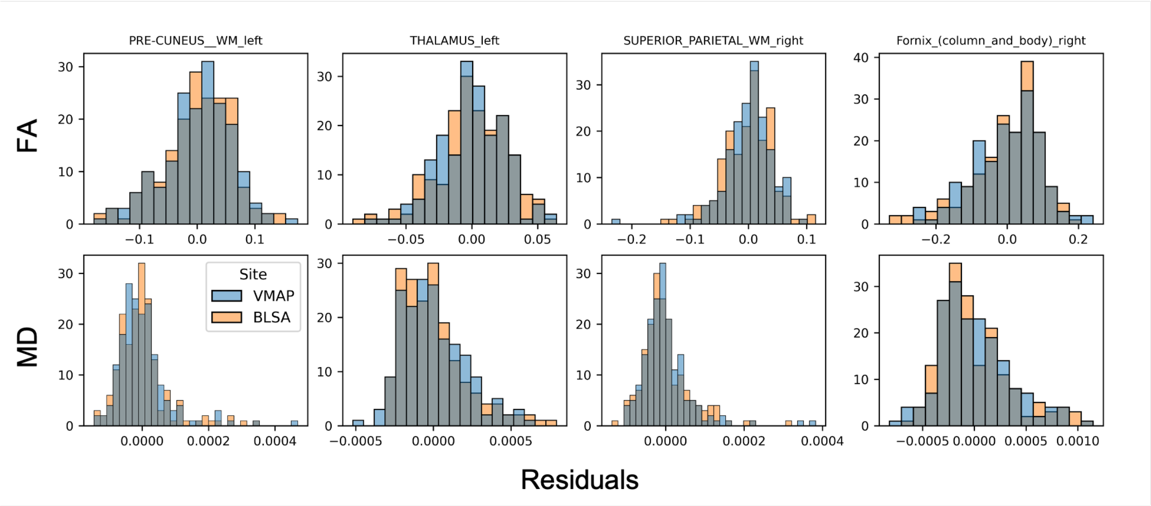

Figure S2. ComBat residuals for different ROIs appear evenly distributed across both sites for the silver standard. We note that the slightly heavy tails for DTI data may be a factor in the reliability of ComBat in situations less like the silver standard.

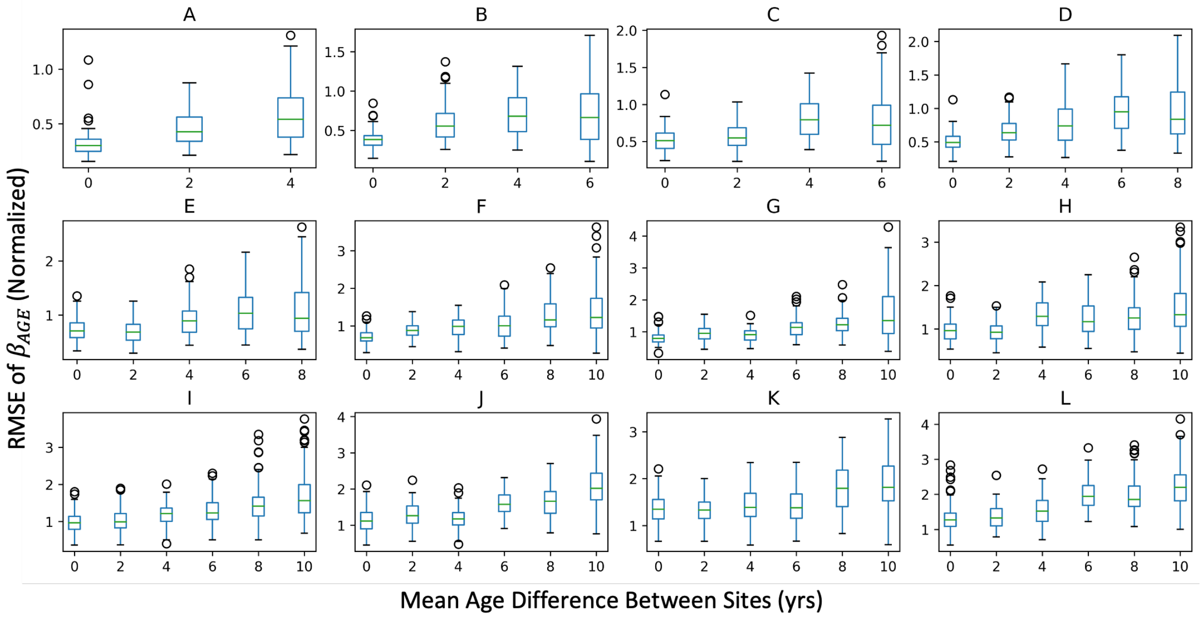

Figure S3. RMSE for standardized β_{AGE} of mean MD vs age compared to the silver standard for sample sizes of (A) $N = 306$, (B) $N = 288$, (C) $N = 270$, (D) $N = 252$, (E) $N = 234$, (F) $N = 216$, (G) $N = 198$, (H) $N = 180$, (I) $N = 162$, (J) $N = 144$, (K) $N = 126$, (L) $N = 108$. Covariate shift does not seem to have a definitive threshold at which the error in estimation of β_{AGE} is much larger compared to the respective experimental run with no covariate shift. For an all-encompassing threshold ambiguous to the size of N, we suggest a maximum covariate shift of 2 years between sites because a covariate shift of either 4 or 6 years increases the error in estimation of β_{AGE} depending on N. Only experimental permutations that have an imbalance ratio of 10:10 were considered.

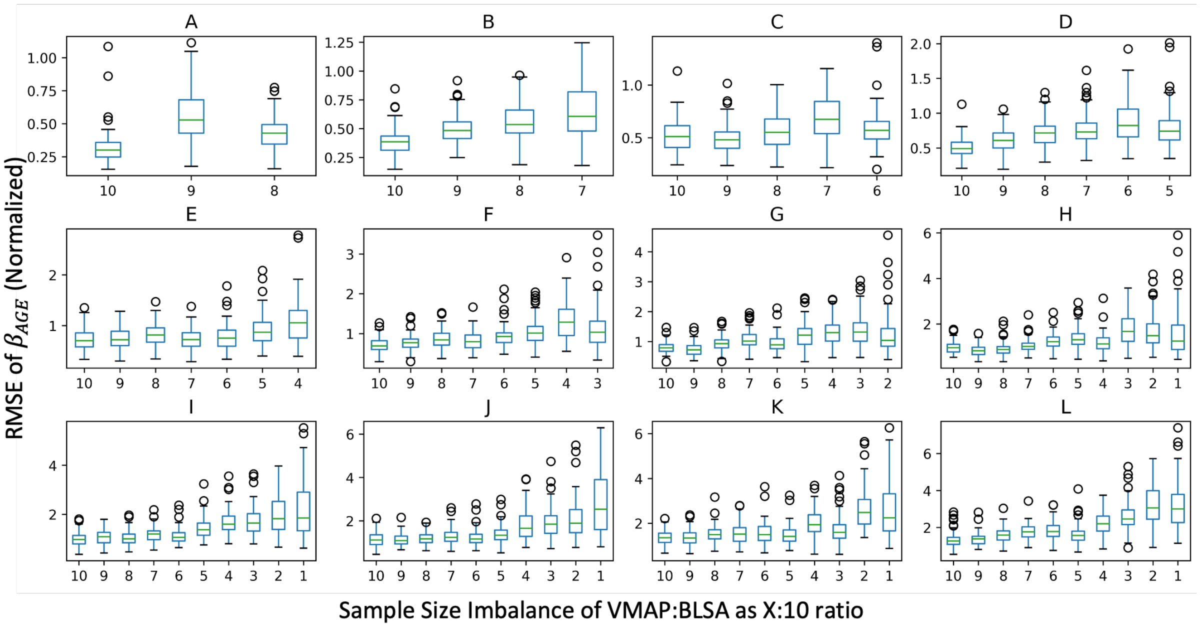

Figure S4. Sample size imbalance alone does not substantially affect estimation of β_{AGE} for mean MD harmonization, only at smaller N does it appear to have an effect. However, this is likely due to the small sample size at VMAP. Only experimental permutations that have a covariate shift of 0 years were considered. A-L are the same N as Fig. 5.

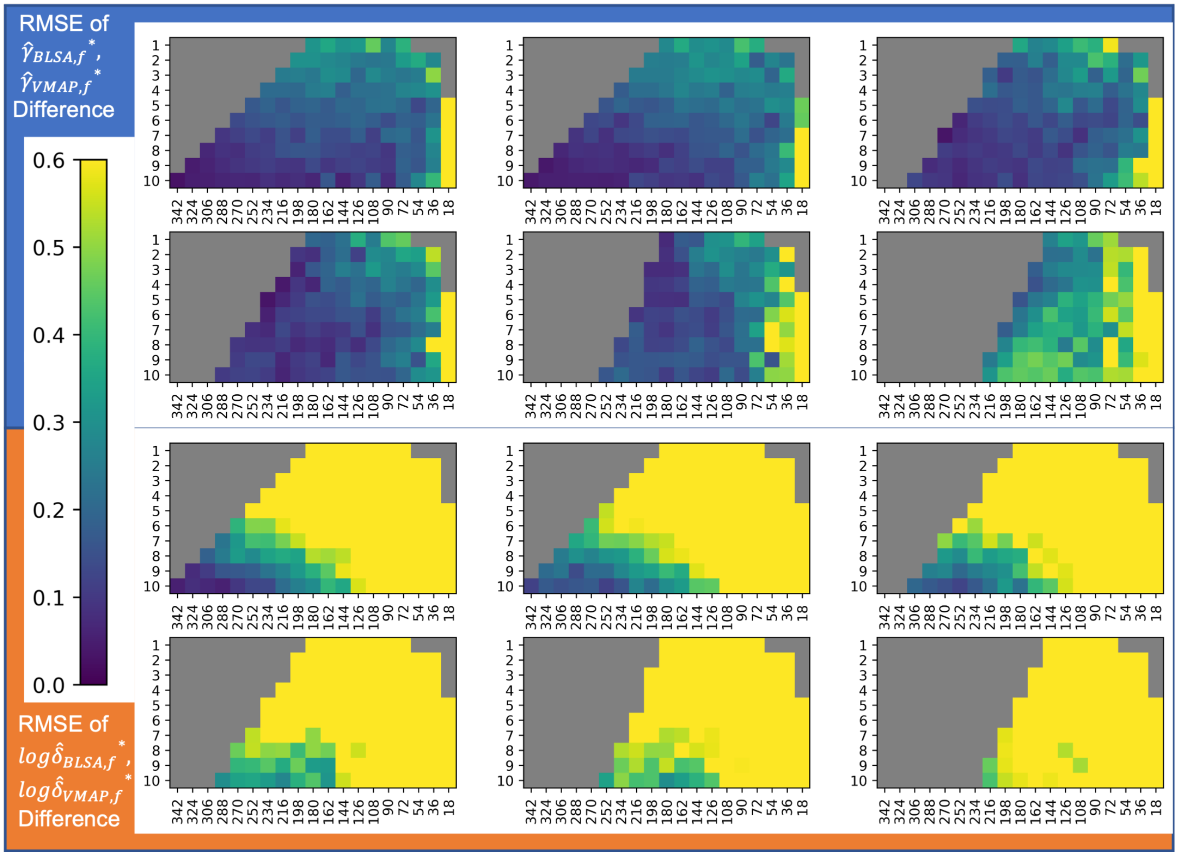

Figure S5. RMSE for $\hat{\gamma}^*_{BLSA,f} - \hat{\gamma}^*_{VMAP,f}$ of experimental runs compared to the silver standard averaged across ROIs shows a similar threshold for stability of around $N \geq 252$ as the lone RMSE of $\hat{\gamma}^*_{sf}$ for both sites. However, the stability of the difference for $log\hat{\delta}^*_{BLSA,f} - log\hat{\delta}^*_{VMAP,f}$ shows a looser threshold of $N \geq 252$ compared to the lone RMSE of $log\hat{\delta}^*_{sf}$ for both sites for mean MD harmonization.

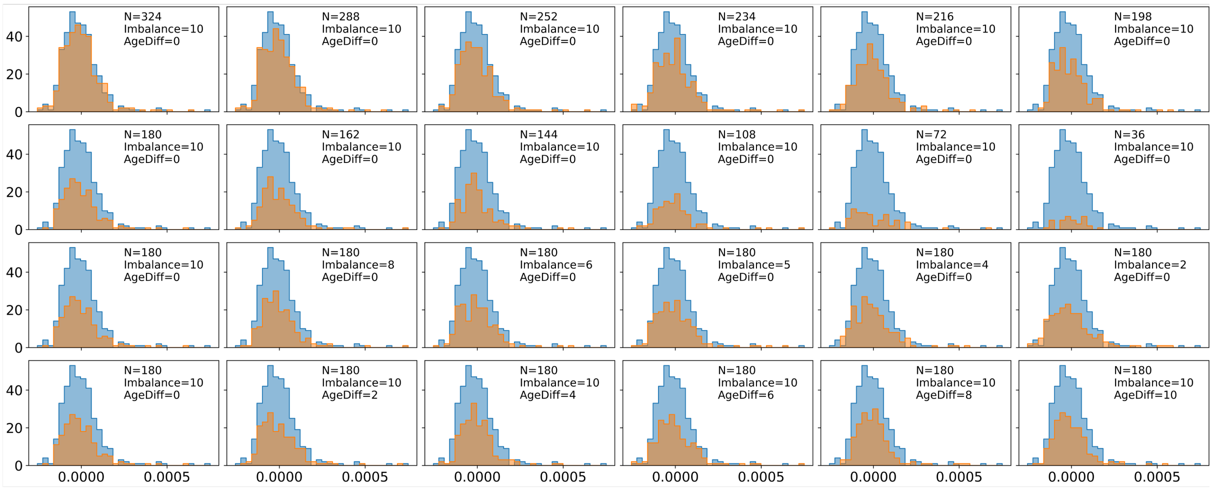

Figure S6. The residuals from the ComBat model for the silver standard (blue, N=358) do not adhere to the assumption of normality given the heavy right tail. As we step further from the silver standard in terms of sample size, imbalance, and covariate shift, we expect the residuals to become even less normally distributed if the assumption of residual normality directly impacts the error in β_{AGE} for experimental runs (blue). Decreasing sample size does not appear to consistently lessen the tail of the residual distributions. The residuals plotted above are for the left genu of the corpus callosum for mean MD harmonization.

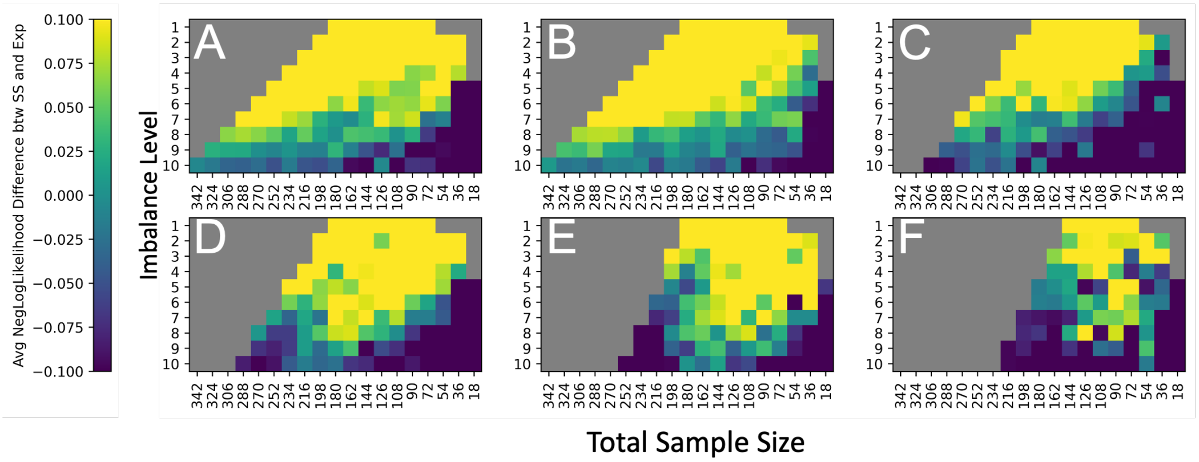

Figure S7. The average negative log likelihood that the residual distribution follows a normal distribution (with mean and standard deviation estimated from the residual distribution) decreases as we decrease in sample size, and is smallest when the sample size is less than 72, suggesting that the residual distributions are more normal at low sample sizes for mean MD harmonization. This contrasts with the increasing error of β_{AGE} with decreasing sample size, suggesting that looking at the distribution of the assumptions alone cannot indicate if ComBat is appropriate for removing site biases of the given input cohort. Thus, we suggest the bootstrapping methodology to determine reliability of ComBat for site bias removal. Difference of average negative log likelihoods for residual distributions between experimental runs and the silver standard negative log likelihoods (averaged across all ROIs). Slices along the covariate shift axis are plotted similarly to Fig. 2.

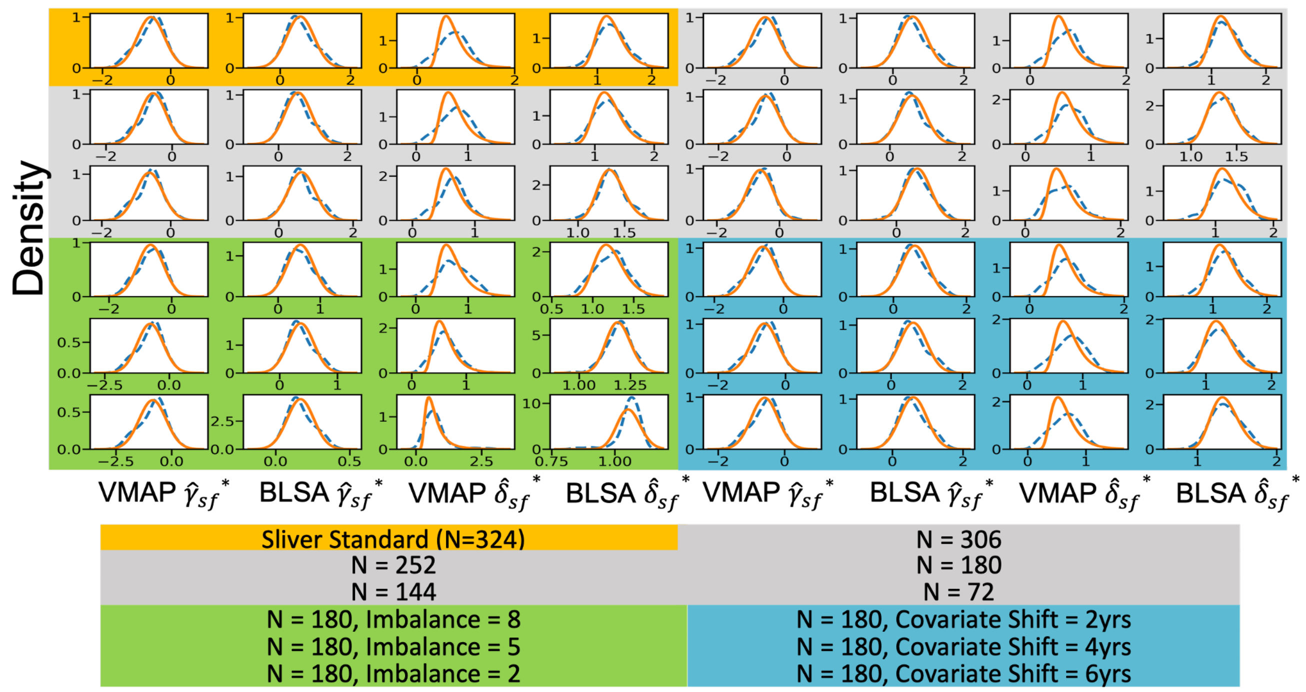

Figure S8. Despite changes in the experimental parameters, the empirical distributions (dotted blue) of the $\hat{\gamma}_{sf}{}^*$ and $\hat{\delta}_{sf}{}^*$ parameters for both sites in MD harmonization often follow the respective estimated distributions as similarly as they do for the silver standard. This adherence to the assumptions on ComBat does not correlate with the respective increasing error in estimates of β_{AGE} for experimental iterations that are further from the silver standard along the experimental axes. $\hat{\gamma}_{sf}{}^*$ is assumed to have a normal distribution per site $\hat{\delta}_{sf}{}^*$ is assumed to have an inverse gamma distribution.

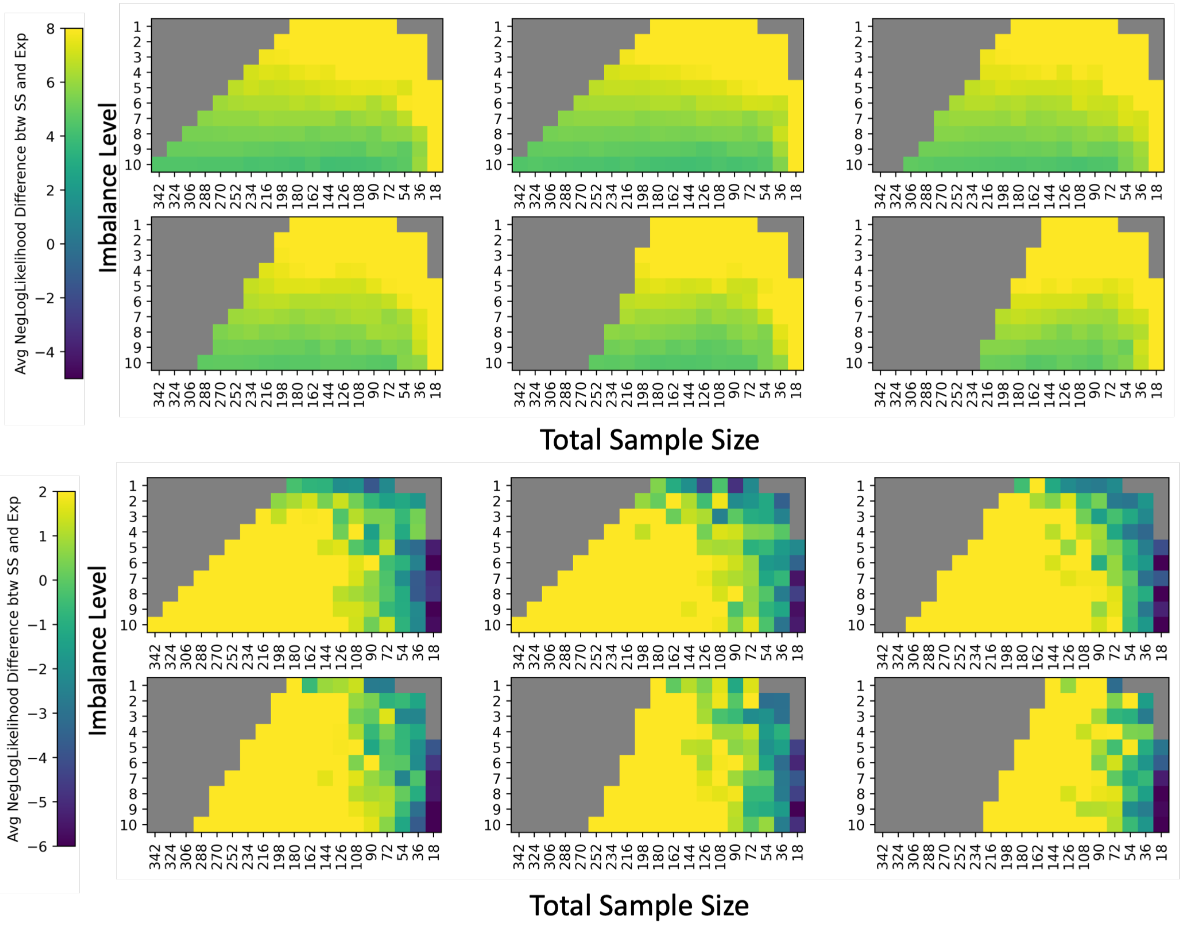

Figure S9. Similar to the ComBat model residuals, the VMAP average negative log likelihoods of the $\hat{\gamma}_{sf}{}^{*}$ estimates (top) for a normal distribution and $\hat{\delta}_{sf}{}^{*}$ estimates (bottom) for an inverse gamma distribution of experimental runs compared to the respective silver standard values do not correlate with the error trends for β_{AGE} in mean MD harmonization. This suggests that we cannot look at the distributions of $\hat{\gamma}_{sf}{}^{*}$ and $\hat{\delta}_{sf}$ to examine whether the input cohort is suitable for ComBat harmonization based on the premise of a violation of ComBat assumptions.

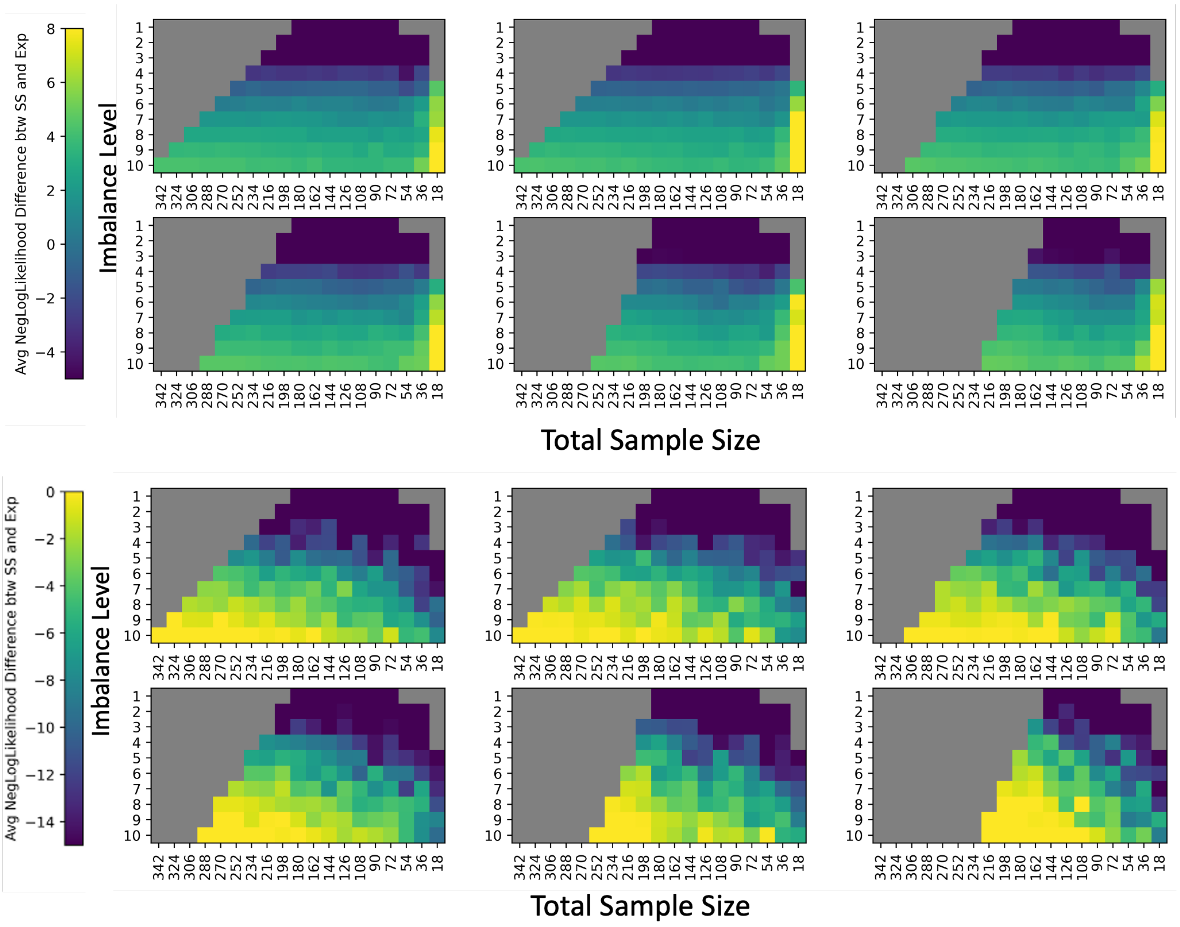

Figure S10. BLSA average negative log likelihoods for $\hat{\gamma}_{sf}^{*}$ (top) and $\hat{\hat{\delta}}_{sf}^{*}$ (bottom) compared to the respective silver standard values do not correlate with the error trends for β_{AGE} in mean MD harmonization.

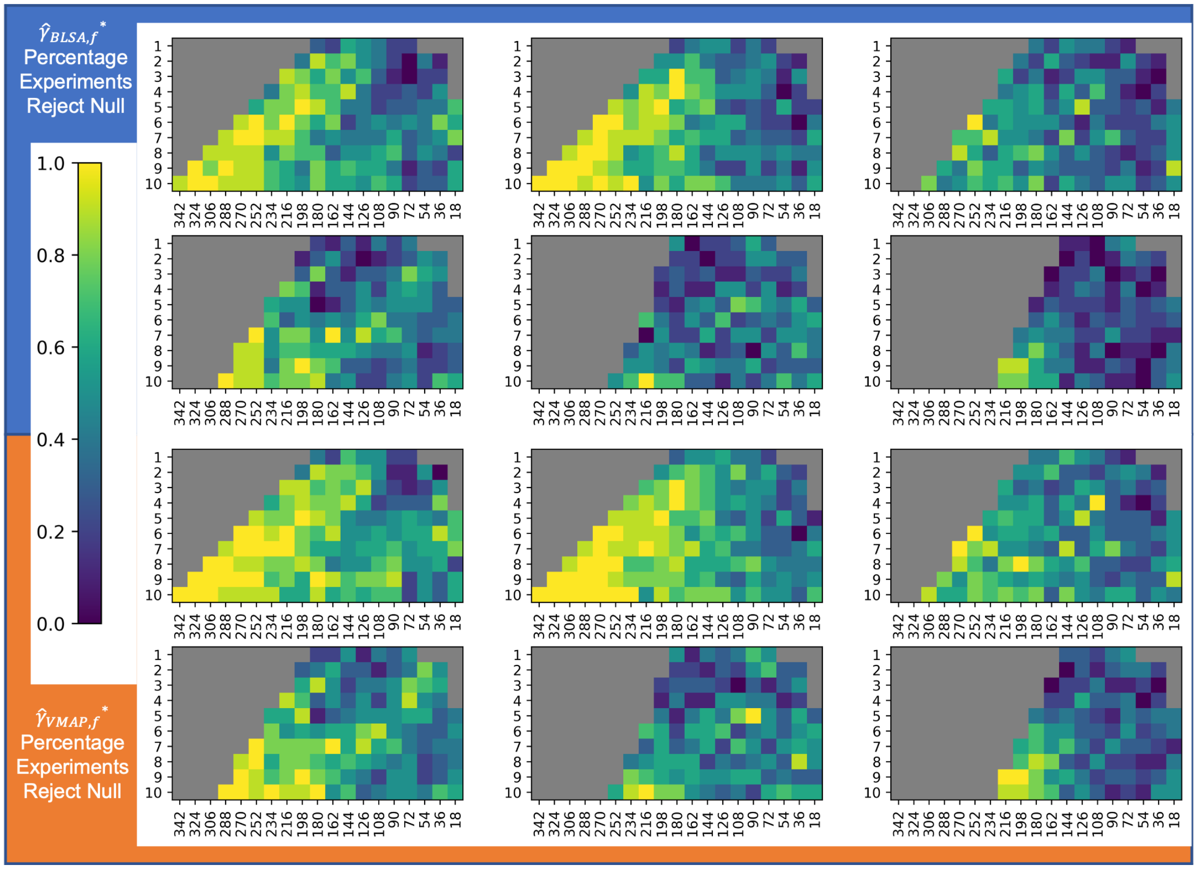

Figure S11. The Anderson-Darling test results suggest that the increase in error for β_{AGE} , and thus the stability of ComBat, cannot be assessed by a decrease in normality of $\hat{\gamma}^{*}_{sf}$ for mean MD harmonization.
